# The Network Survival Method for Estimating Adult Mortality: Evidence From a Survey Experiment in Rwanda

**DOI:** 10.1007/s13524-017-0594-y

**Published:** 2017-07-24

**Authors:** Dennis M. Feehan, Mary Mahy, Matthew J. Salganik

**Affiliations:** 1 Department of Demography, University of California, Berkeley, Berkeley, CA USA; 2 Joint United Nations Programme on HIV/AIDS (UNAIDS), Geneva, Switzerland; 3 Department of Sociology and Office of Population Research, Princeton University, Princeton, NJ USA

**Keywords:** Adult mortality, Social networks, Sampling, Demographic and Health Surveys, Survey experiment

## Abstract

Adult death rates are a critical indicator of population health and well-being. Wealthy countries have high-quality vital registration systems, but poor countries lack this infrastructure and must rely on estimates that are often problematic. In this article, we introduce the *network survival method*, a new approach for estimating adult death rates. We derive the precise conditions under which it produces consistent and unbiased estimates. Further, we develop an analytical framework for sensitivity analysis. To assess the performance of the network survival method in a realistic setting, we conducted a nationally representative survey experiment in Rwanda (*n* = 4,669). Network survival estimates were similar to estimates from other methods, even though the network survival estimates were made with substantially smaller samples and are based entirely on data from Rwanda, with no need for model life tables or pooling of data from other countries. Our analytic results demonstrate that the network survival method has attractive properties, and our empirical results show that this method can be used in countries where reliable estimates of adult death rates are sorely needed.

**Electronic supplementary material:**

The online version of this article (doi:10.1007/s13524-017-0594-y) contains supplementary material, which is available to authorized users.

## Introduction

Adult death rates are a critical indicator of population health and well-being. In developed countries, a variety of legal, medical, and financial systems ensure that virtually every death is recorded in a vital registration system. These vital registration systems enable researchers to produce high-quality estimates of adult death rates by age and sex. Most developing countries, on the other hand, are victims of the *scandal of invisibility*: because administrative systems that reliably produce death certificates are lacking, most adults die without ever having their deaths formally recorded (AbouZahr et al. 2015; Mikkelsen et al. 2015; Setel et al. 2007). The scandal of invisibility is, unfortunately, vast: Mikkelsen et al. (2015) estimated that two-thirds of worldwide deaths are never formally recorded.

The long-term solution to the scandal of invisibility is for all countries to develop effective vital registration systems. Progress on this front, however, has been very slow: Mikkelsen et al. (2015) estimated that between 2000 and 2012, the percentage of deaths registered worldwide increased from 36 % to only 38 %. Because of the absence of high-quality vital registration data in developing countries, researchers have worked on the problem of estimating adult death rates for decades. Unfortunately, this problem has proven to be extremely difficult. In the meantime, critical questions about science and policy in the world’s poorest countries continue to go unanswered.

This article helps address the scandal of invisibility by developing and testing the *network survival method*, a new survey-based method for estimating adult mortality. Roughly, this new method generalizes the *sibling survival method*, which is the survey-based approach that is most widely used today. Whereas the sibling survival method collects information about the deaths of siblings of respondents, the network survival method collects information about deaths in a wider social network around each respondent. The generalization dramatically increases the amount of information collected from each respondent, but it also introduces a variety of complexities that our methodology addresses. Because the network survival method uses data that could be collected in a standard household survey—the kind of surveys routinely fielded in most developing countries—it could potentially be deployed in developing countries around the world.

## Background

### Estimating Death Rates

The *death rate* is the number of deaths that occur in a group, relative to the group’s exposure to the possibility of dying. Mathematically, for a demographic group α (for example, women aged 45–49 in 2011), the death rate can be written as follows:

1 $$ {M}_{\upalpha}=\frac{D_{\upalpha}}{N_{\upalpha}}, $$

where *D*_α_ is the number of deaths, and *N*_α_ is the amount of exposure to demographic group α. Death rates are a type of occurrence-exposure rate.

Adult death rates are difficult to estimate from a survey for two main reasons (Timaeus 1991). First, surveys typically ask respondents to report about themselves; for example, a survey might ask respondents to report their age, education, or income. This approach is not possible for deaths because people who have died cannot be interviewed. Second, adult deaths are quite rare; even in poor countries, death rates lower than 10 per 1,000 are not unusual for some age ranges. Rare events are difficult to study using standard survey techniques because they require very large samples to yield estimates that are precise enough to be useful (Kalton and Anderson 1986). Any survey-based approach to estimating adult death rates will have to overcome these two primary obstacles.

If death rates are difficult to estimate from surveys, why focus on survey-based approaches at all? We believe that surveys offer the best hope for immediate, global, and sustained progress, as has been illustrated by the progress that has been made using surveys to estimate other critical demographic quantities, such as fertility and child mortality. In countries that lack good vital registration systems, fertility rates and child mortality were once as poorly understood as adult mortality is now. Today, though, even the world’s poorest countries have high-quality estimates of fertility and child mortality rates. Researchers had to develop new methods to estimate these quantities from household surveys (Hill and Choi 2004; Timaeus 1991), and these methods had to be tested and refined in realistic field conditions until they were able to be deployed at a global scale—first with the World Fertility Survey Program, and now through the massive, internationally coordinated, Demographic and Health Survey (DHS) program and the Multiple Indicator Cluster Survey program (Corsi et al. 2012; Fabic et al. 2012; Hancioglu and Arnold 2013; Hill et al. 2007). In fact, because of these earlier efforts, high-quality household surveys are already being regularly conducted in countries without vital registration systems. This survey infrastructure can be harnessed to estimate adult mortality.

### Sibling Survival Method

Previous research on adult mortality estimation has considered many different strategies for collecting information about deaths, including surveys, prospective or cohort designs, incomplete sources of death certificates, one or many censuses, and historical records. Other researchers have provided more complete overviews of mortality estimation (see, e.g., Bradshaw and Timaeus 2006; Gakidou et al. 2004; Hill 2001, 2003; Hill et al. 2005, 2007; Reniers et al. 2011; Timaeus 1991; United Nations 1983). In this article, we focus on survey-based techniques because they are most relevant to our new estimator. Many survey-based approaches can be used to estimate death rates, but the most common is the *direct sibling survival method* (Rutenberg and Sullivan 1991),^1^ which requires collecting sibling histories: each respondent is asked to enumerate her or his siblings and then to provide each sibling’s birthday, survival status, and date of death (when applicable).

The direct sibling survival method seems like a promising way to overcome the two fundamental challenges in estimating death rates from surveys: (1) because respondents report about their siblings, it is possible to learn about people who have died; and (2) because respondents typically have multiple siblings, each interview produces information about more than one person, increasing the effective size of the sample. As a part of the DHS program, sibling histories have been collected in more than 150 surveys from dozens of countries across the developing world (Corsi et al. 2012; Fabic et al. 2012). Nonetheless, relatively few researchers have made use of these DHS sibling histories to study adult mortality (Gakidou et al. 2004; Reniers et al. 2011). For example, despite the fact that very little is known about adult mortality in sub-Saharan Africa (Setel et al. 2007), only a handful of studies have tried to use the DHS sibling histories to construct estimates of recent trends in adult mortality (Masquelier et al. 2014; Obermeyer et al. 2010; Rajaratnam et al. 2010; Reniers et al. 2011; Timaeus and Jasseh 2004; Wang et al. 2013).

DHS sibling histories may have been relatively underused for two reasons. First, surveys with typical DHS sample sizes—between 5,000 and 30,000 respondents (Corsi et al. 2012)—cannot be used to produce timely direct estimates of age- and sex-specific death rates because the sampling variation from the direct sibling survival estimator is too large (Hill et al. 2006; Stanton et al. 2000; Timaeus and Jasseh 2004). Instead, researchers have had to resort to a combination of pooling data across countries and across time, smoothing regressions, and model life tables to estimate adult mortality from DHS sibling histories (Masquelier et al. 2014; Obermeyer et al. 2010; Rajaratnam et al. 2010; Reniers et al. 2011; Timaeus and Jasseh 2004; Wang et al. 2013). This need to smooth the raw data requires researchers to make several difficult-to-verify assumptions, reducing the appeal of producing estimates based on sampled data (Masquelier 2013).

The second reason why DHS sibling histories may be relatively underused is the methodological uncertainty about how sibling histories should be analyzed. Several common methodological concerns have emerged from research about the sibling histories: (1) there is no way to learn about *sibships* (sets of people who are siblings) that have no survivors left to be sampled by the survey; (2) more generally, sibships with more survivors are more likely to be sampled by the survey, potentially biasing estimates if sibship size and mortality are correlated (Gakidou and King 2006; Gakidou et al. 2004; Graham et al. 1989; Masquelier 2013; Reniers et al. 2011; Trussell and Rodriguez 1990); (3) there are many ways that respondents’ reports about their siblings may not be accurate—for example, respondents may omit some siblings from their survey reports, and if the tendency to omit a sibling is correlated with the chances that the sibling is alive, then this may introduce bias into the resulting estimates (Helleringer et al. 2013, 2014a, b; Masquelier and Dutreuilh 2014; Merdad et al. 2013); and (4) the respondent is, by definition, alive, making it unclear whether the respondent’s experience should be included or omitted from the death rate estimates (Masquelier 2013; Reniers et al. 2011).

Uncertainty about these methodological issues has not been resolved. For example, Gakidou and King (2006) proposed a solution to address the potential correlation between sibship size and mortality, but the method has proven to be controversial in practice (Masquelier 2013). Subsequent studies have therefore been divided: one group has applied the Gakidou-King selection bias adjustments (Kassebaum et al. 2014; Rajaratnam et al. 2010; Wang et al. 2013), while another has not (Masquelier et al. 2014; Moultrie et al. 2013; Reniers et al. 2011).

To conclude, the direct sibling survival method is a promising approach to overcoming the two main challenges that must be faced to estimate death rates from a survey: (1) it enables researchers to learn about people who died, and (2) it enables researchers to learn about more than one person from each interview. Unfortunately, in practice, the direct sibling survival method has two big disadvantages. First, this method cannot typically be used to produce direct estimates of death rates because the sampling variation of direct estimates is too large. Second, the sibling survival method is clouded by several potential sources of bias. It is not clear precisely what effect these potential biases might have on sibling survival estimates, or how these potential biases might interact with one another.

## The Network Survival Method

The network survival method can be seen as a generalization of the direct sibling survival method. Whereas the direct sibling survey method collects information about mortality in sibling networks, the network survival method collected information about mortality in *any* type of network in which respondents are embedded.

The network survival method collects two types of information about survey respondents’ personal networks. First, respondents are asked about their connections to people who died: for example, “How many people do you know who died in the previous 12 months?,” where “know” could be replaced with other types of social relationships, as we discuss later. Similar to a sibling history, respondents are asked to enumerate each person who died and to provide additional information, such as age and sex, about each one. Second, unlike the sibling survival method, respondents are also asked about their connections to several different groups whose total size is known: for example, “How many policemen do you know?,” where the number of policemen is available from administrative records or estimated from a survey. This information about connections to groups of known size is used to estimate the total size of respondents’ personal networks, and this approach has been used as part of the network scale-up method (Bernard et al. 2010; Feehan and Salganik 2016a; Killworth et al. 1998b).

Asking survey respondents to report about the members of their personal networks helps resolve both of the major difficulties in estimating death rates from a survey. Because respondents report about others, it is possible to learn about people who have died, even though the people who died cannot be interviewed directly. And, because respondents are asked to report about all the people in their personal networks, researchers obtain information about much more than just one person from each interview, increasing the effective sample size.

In the remainder of this section, we turn to a more detailed description of how the network survival method estimates death rates. Our focus will be on describing the main ideas behind the new estimator; Online Resource 1 (sections A–I) provides proofs and further technical details.

### Estimating the Number of Deaths, *D*_α_

**Fig. 1:**
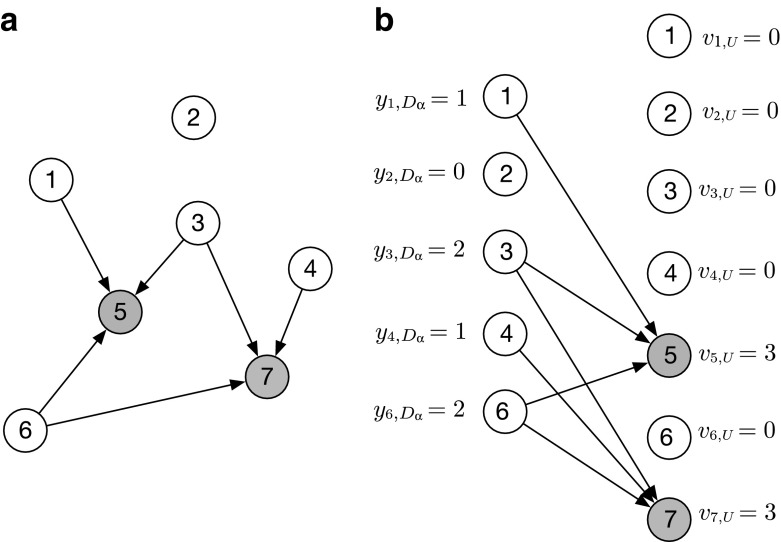
Panel a shows a population of seven people, two of whom have died (*shown in gray*). A directed edge *i* → *j* indicates that *i* counts *j* as having died when answering the question, “How many people do you know who have died in the past 12 months?” Panel b shows the same population but redrawn so that each person now appears twice: as someone who reports (*left*) and as someone who could be reported about (*right*). People who have died cannot report (they cannot be interviewed). This figure depicts detailed individual reports *i* → *j*; but in practice, reports are not typically collected at that level of detail (i.e., we typically would know that person *i* reports one death, but not that the death was specifically person *j*). Fortunately, the identity in Eq. (3) requires estimates of aggregate quantities, so this level of detail is not required

The numerator of a death rate is the number of deaths in demographic group α (*D*_α_).^2^ Estimating this quantity from network reports is complex because each individual death could be reported multiple times (or not at all). We must therefore convert respondents’ *reports* about deaths into an estimate for the *number* of deaths in the population. To make this conversion, we use the network reporting framework (Feehan 2015; Feehan and Salganik 2016a), which is illustrated in Fig. 1. Panel a of the figure depicts individuals in a population who have been asked to report which of their personal network members have died in the past 12 months. Each directed arrow *i → j* indicates that *i* reports that *j* has died. Panel b presents the same information, but this information is rearranged so that the people who report are on the left, and the people who could be reported about are on the right. Note that living people can both report and be reported about, since a living person can be erroneously reported as dead.

Using this framework, we can create a reporting identity:

2 $$ \mathrm{total}\ \mathrm{number}\ \mathrm{of}\ \mathrm{reports}\ \mathrm{about}\ \mathrm{death}\mathrm{s}=\mathrm{number}\ \mathrm{of}\ \mathrm{death}\mathrm{s}\times \mathrm{average}\ \mathrm{reports}\ \mathrm{per}\ \mathrm{death}. $$

Rearranging Eq. (2) yields

3 $$ \mathrm{number}\ \mathrm{of}\ \mathrm{death}\mathrm{s}=\frac{\mathrm{total}\ \mathrm{number}\ \mathrm{of}\ \mathrm{reports}\ \mathrm{about}\ \mathrm{death}\mathrm{s}}{\mathrm{average}\ \mathrm{reports}\ \mathrm{per}\ \mathrm{death}}. $$

The identity in Eq. (3) reveals that we can estimate the number of deaths from respondents’ reports by estimating (1) the total number of reports about deaths that would be collected if we interviewed everyone, and (2) the average number of reports per death. A helpful way to think about the identity in Eq. (3) is that it clarifies the appropriate way to adjust reports of deaths in order to avoid overcounting the same death multiple times.

Mathematically, the identity in Eq. (3) can be written as

4 $$ {D}_{\upalpha}=\frac{y_{F,{D}_{\upalpha}}}{\upsilon_{U,F}/{D}_{\upalpha}}, $$

where *U* is the entire population; *F* is the *frame population* (the set of people on the sampling frame; in many cases, this will be all adults); ${y}_{F,{D}_{\upalpha}}={\sum}_{i\in F}{y}_{i,{D}_{\upalpha}}$ is the number of deaths in demographic group α that would be reported if everyone in the frame population *F* was interviewed (i.e., in a census); and *υ*_*U* , *F*_ = ∑_*j* ∈ *U*_ *υ*_*j* , *F*_ is the total visibility of all deaths (i.e., the number deaths in the entire population that would be reported if everyone in the frame population was interviewed).

There turns out to be a practical problem with trying to develop an estimator from the identity in Eq. (4): *υ*_*U* , *F*_ is the number of times anyone in the population would be reported as dead, but it is much more feasible to estimate the number of times that anyone who actually died would be reported as dead. Therefore, we assume that respondents do not incorrectly report that someone died when in fact she did not. In this case, we say that there are no *false positive* reports. (Later in the article, we develop a full framework for sensitivity analysis that shows exactly how estimates can be affected by violations of this assumption.)

If there are no false positive reports, then *υ*_*j* , *F*_ = 0 for all people *j* who are alive and thus ${\upsilon}_{U,F}={\upsilon}_{D_{\upalpha},F}$. We can then rewrite Eq. (4) as follows:

5 $$ {D}_{\upalpha}=\frac{y_{F,{D}_{\upalpha}}}{{\overline{\upsilon}}_{D_{\upalpha},F}}, $$

where ${\overline{\upsilon}}_{D_{\upalpha},F}={\upsilon}_{D_{\alpha },F}/{D}_{\alpha }$ is the *visibility* of deaths: the average number of times that each death in group α would be reported if everyone in the frame population was interviewed.

The network survival estimate for the number of deaths in demographic group α (*D*_α_) is based on Eq. (5). The numerator of Eq. (5), ${y}_{F,{D}_{\upalpha}}$, is the total reported connections to deaths. This quantity can be estimated from the data we collect about respondents’ connections to people who have died using a standard Horvitz-Thompson approach:

6 $$ {\widehat{y}}_{F,{D}_{\upalpha}}=\sum_{i\in s}{y}_i,{D}_{\alpha }/{\uppi}_i, $$

where π_*i*_ is the probability that respondent *i* was included in our sample. π_*i*_ is typically known from the survey’s sampling design. See Result B.1 in Online Resource 1 for a formal statement and proof.

The denominator of Eq. (5) is the visibility of deaths, ${\overline{\upsilon}}_{D_{\upalpha},F}$. This quantity is more difficult to estimate. There are many possible approaches, but we propose using the estimated average personal network size of survey respondents in demographic group α to estimate the visibility of deaths in demographic group α. (We describe how to estimate personal network sizes later.) For example, our approach is to assume that the visibility of deaths among women aged 45–54 (i.e., the number of times each of these deaths could be reported) is the same as the personal network size of women in the frame population aged 45–54. Using respondents’ average personal network size to estimate the visibility of deaths will be exactly correct if (1) people who die in group α have personal networks that are the same size, on average, as survey respondents in group α (the *decedent network assumption*); and (2) survey respondents are perfectly aware of and report all the deaths in their personal networks (the *accurate reporting assumption*). (See Result B.2 in Online Resource 1 for a formal statement and proof.) These are both strong assumptions; for example, people who die might have smaller personal networks if they experience an illness that reduces the size of their personal networks in the time leading up to death. Later, we develop a full framework for sensitivity analysis that shows exactly how estimates are affected by violations of these assumptions.

### Estimating the Average Personal Network Size of Group α, ${\widehat{\overline{d}}}_{F\boldsymbol{\upalpha}, F}$

To estimate the average personal network size of respondents in demographic group α, we adapt the *known population method* (Killworth et al. 1998a), which asks respondents questions about their connections to groups of known size (e.g., “How many policemen do you know?”); intuitively, the more connections a respondent reports to policemen, the bigger we estimate her personal network to be. Respondents are typically asked about their connections to about 20 different groups of known size, and the results are combined using the known population estimator (Bernard et al. 2010; Feehan and Salganik 2016a; Killworth et al. 1998a).

The known population estimator was designed to estimate personal network sizes for individual respondents. Fortunately, we have a slightly easier problem: estimating the average personal network size for a group of people. Therefore, in Online Resource 1, we derive an adapted estimator for the average network size of respondents in a particular demographic group α. The main advantage of our adapted approach is that it requires slightly weaker conditions than the traditional known population estimator. The adapted known population estimator is

7 $$ {\widehat{\overline{d}}}_{F_{\upalpha},F}=\frac{\sum_{i\in {s}_{\alpha }}{\sum}_j{y}_{i,{A}_j}/{\uppi}_i}{\sum_j{N}_{A_j}}\frac{N_F}{N_{F_{\alpha }}}, $$

where ${\overline{d}}_{F_{\upalpha},F}={d}_{F_{\alpha },F}/{N}_{F_{\alpha }}$ is the average number of network connections between frame population members in demographic group α (*F*_α_) and all the members of the frame population (*F*); *N*_*F*_ is the size of the frame population; ${N}_{F_{\upalpha}}$ is the number of frame population members who are also in demographic group α; *s*_α_ is the subset of survey respondents in demographic group α; *j* ∈ {1, . . . , *J*} indexes the groups of known size; ${y}_{i,{A}_j}$ is the number of connections that respondent *i* reports to group of known size *A*_*j*_; and ${N}_{A_j}$ is the size of the *j*th group of known size. See Result A.1 in Online Resource 1 for a formal statement and proof.

Combining the estimator for the number of reported deaths in group α (Eq. (6)) with the estimator for the personal network size of survey respondents in group α (Eq. (7)) yields our estimator for the number of deaths in group α:

8 $$ {\widehat{D}}_{\upalpha}=\frac{{\widehat{y}}_{F,{D}_{\upalpha}}}{{\widehat{\overline{d}}}_{F_{\upalpha},F}}. $$

See Result B.3 in Online Resource 1 for a formal statement and proof.

### Estimating the Exposure, *N*_α_

To convert the estimated total number of deaths into a death rate, we need to estimate the amount of exposure *N*_α_. If the sampling frame includes all adults, then

9 $$ {N}_{\upalpha}={N}_{F_{\alpha }}, $$

and we say the frame population is *complete* for α. When the frame population is complete for α, researchers can use information from the sample design to estimate *N*_α_:

10 $$ {\widehat{N}}_{\upalpha}=\sum_{i\in {s}_{\alpha }}\frac{1}{\uppi_i}. $$

If the sampling frame is not complete and if high-quality estimates for the exposure *N*_α_ are available from other sources, then researchers can use the alternative approaches described in Online Resource 1, Result B.4.

### Putting It All Together to Estimate Death Rates, ${\widehat{\mathrm{M}}}_{\boldsymbol{\upalpha}}$

Combining the estimator for the number of deaths (Eq. (8)) and the estimator for the exposure (Eq. (10)), and simplifying, leads to the *network survival estimator* for the death rate in group α:

11 $$ {\widehat{M}}_{\upalpha}=\frac{{\widehat{y}}_{F,{D}_{\upalpha}}}{{\widehat{\overline{d}}}_{F_{\upalpha},F}}\frac{1}{{\widehat{N}}_{F_{\upalpha}}}. $$

See Result B.5 in Online Resource 1 (section B) for a formal statement and proof.

## The Network Survival Method in Rwanda

The preceding arguments and the proofs in Online Resource 1 show that the network survival method has attractive theoretical properties. They tell us little, however, about how the method actually works in practice. The ideal way to assess any new method is to use it in a situation like the ones where it will be used in practice *and* where it can be validated. These two conditions, unfortunately, are rarely satisfied together. Typically, we can test a new method in either a realistic situation or in a situation where it can be validated. For this study, we chose to test the network survival method in a realistic situation: a large household survey in Rwanda, a country without a high-quality vital registration system. This study alone, therefore, cannot be used to fully assess the network survival method. However, neither could a study using the network survival method in the United States, a setting with a high-quality vital registration system but which is unlike countries where the network survival method will typically be used. Ultimately, we think that empirical assessment of the network survival method must involve both studies in realistic field situations and studies where estimates can be validated against gold standard measures.

The network survival method can be used to collect reports about people connected to respondents in almost any way. Therefore, we had to decide who we would ask respondents to report about. In other words, we had to choose the tie definition that would be used in our study; this terminology comes from the social networks literature, where a connection between nodes in a network is called a *tie*.

Because people are embedded in many different personal networks—friendship networks, family networks, occupational networks, and so forth—the ability to choose a tie definition makes the network survival method very flexible. Further, we expect that the choice of tie definition will have implications for both sampling and nonsampling error because it implies a trade-off between the quality and quantity of information collected in each interview (Feehan et al. 2016). Roughly, we expect that using a weaker tie definition will collect more, noisier information per interview. Using a stronger tie definition, on the other hand, could produce more accurate information but about a small number of other people. Obviously, researchers would like to choose a tie definition that would minimize total error (i.e., sampling error plus nonsampling error). Because no network survival data has been collected previously, we had no way to assess this trade-off empirically before embarking.

Therefore, we conducted a survey experiment that randomized respondents to report about one of two different types of personal network: (1) half of our sample reported a relatively weak tie network—their *acquaintance network*; (2) the other half of the sample reported about a relatively strong tie network—their *meal network* (Table 1). The acquaintance tie definition has been used in all previous network scale-up studies (Bernard et al. 2010), and our study was the first to use the meal definition, which we devised and refined in collaborations with local experts in Rwanda. We pilot tested both definitions to ensure that they were appropriate in Rwanda. Overall, this survey experiment enables us to better understand this key aspect of the method.

**Table 1:** The two definitions of a personal network connection (or tie) used in this study

Acquaintance (*n* = 2,236)	Meal (*n* = 2,433)
• People of all ages who live in Rwanda	• People of all ages who live in Rwanda
• People the respondent knows, by sight AND name, and who also know the respondent by sight and name	• People the respondent knows, by sight AND name, and who also know the respondent by sight and name
• People the respondent has had some contact with—either in person, over the phone, or on the computer in the previous 12 months	• People the respondent has shared a meal or drink with in the past 12 months, including family members, friends, co-workers, or neighbors, as well as meals or drinks taken at any location, such as at home, at work, or in a restaurant

*Note:* All conditions need to be satisfied for the respondent to consider someone a member of her network.

### Data Collection

Our survey used the same interviewers, data entry protocols, training techniques, and sampling procedures as the 2010 Rwanda DHS. By using the DHS infrastructure, we ensure that our research design can be used in face-to-face surveys in developing countries across the world. Our sample–which was a special survey, distinct from the 2010 Rwanda DHS–was drawn using a stratified, two-stage cluster design, and interviews were conducted between June and August of 2011. The household response rate was 99 %, and the individual response rate was 97 %. The full details of the sampling plan and field procedures are described elsewhere (Rwanda Biomedical Center/Institute of HIV/AIDS et al. 2012). Following the guidelines of the DHS program (ICF International 2012: sec. 1.13.7), we denormalize the sampling weights by using the United Nations Population Division (UNPD) estimates for the size of Rwanda’s population aged 15 and older in 2010 (United Nations 2013). When quantifying the sampling uncertainty in our estimates, we use the rescaled bootstrap to account for our complex sample design (Feehan and Salganik 2016a; Rao and Wu 1988; Rao et al. 1992).

Each sampled household was randomly assigned to one of the two possible definitions of a network, and balance checks show that the randomization was successfully implemented (Feehan et al. 2016). All adults in each household were interviewed. Our choice to interview all adults differs from a typical DHS, which interviews women up to age 50 and men up to age 60; we discuss this difference and its implication for estimates in greater detail in Online Resource 1 (section G). Table 2 shows the known populations that were used to estimate personal network sizes in our study in Rwanda. More information about how these particular known populations were chosen and general advice about choosing known populations can be found elsewhere (Feehan and Salganik 2016a; Feehan et al. 2016; Rwanda Biomedical Center/Institute of HIV/AIDS et al. 2012).

**Table 2:** The known populations used to estimate network sizes in the Rwanda study

Group Name	Size	Source
Priests	1,004	Catholic Church
Nurses or Doctors	7,807	Ministry of Health
Twahirwa^a^	10,420	ID database
Mukandekezi^a^	10,520	ID database
Nyiraneza^a^	21,705	ID database
Male Community Health Worker	22,000	Ministry of Health
Ndayambaje^a^	22,724	ID database
Murekatete^a^	30,531	ID database
Nsengimana^a^	32,528	ID database
Mukandayisenga^a^	35,055	ID database
Widowers	36,147	RDHS (05, 07, 10)
Ndagijimana^a^	37,375	ID database
Bizimana^a^	38,497	ID database
Nyirahabimana^a^	42,727	ID database
Teachers	47,745	Ministry of Education
Nsabimana^a^	48,560	ID database
Divorced Men	50,698	RDHS (05, 07, 10)
Mukamana^a^	51,449	ID database
Incarcerated People	68,000	ICRC 2010 report
Women Who Smoke	119,438	RDHS (05)
Muslim	195,449	RDHS (05, 07, 10)
Women Who Gave Birth in the Last 12 Months	256,164	RDHS (10)

*Note: RDHS* denotes the Rwanda Demographic and Health Survey from the years indicated in parentheses; *ID database* denotes counts of names from the national identity card database; and *ICRC* is the International Committee of the Red Cross.

^a^A Kinyarwanda name.

We had to pay careful attention to constructing the wording of the question that asked respondents to report about deaths. Both tie definitions used in our study in Rwanda were based on interactions (Table 1): (1) contact, for the acquaintance definition, or (2) sharing a meal or drink, for the meal definition. Of course, people who have died cannot continue to interact with others. We therefore expect people who died in the 12 months before a survey to have had fewer total interactions than people who did not. This expected systematic difference is problematic for network survival estimates, which are based on the assumption that the visibility of deaths can be estimated by the personal network size of survey respondents (the *decedent network assumption* in Result B3, Online Resource 1). Thus, we do not want the personal networks of people who died to be smaller, on average, than people who lived. We attempted to circumvent this potential problem in our study by asking respondents to report people who satisfy two conditions: (1) the person died in the 12 months before the interview, and (2) the person shared a meal with the respondent in the 12 months before death. We discuss this choice, its possible effect on estimates, and alternative approaches in Online Resource 1 (section I), which also includes an excerpt of the English translation of the survey instrument. All survey materials, including the original Kinyarwanda instruments, are freely available from the DHS website (Rwanda Biomedical Center/Institute of HIV/AIDS et al. 2012).

### Basic Descriptive Statistics

**Fig. 2:**
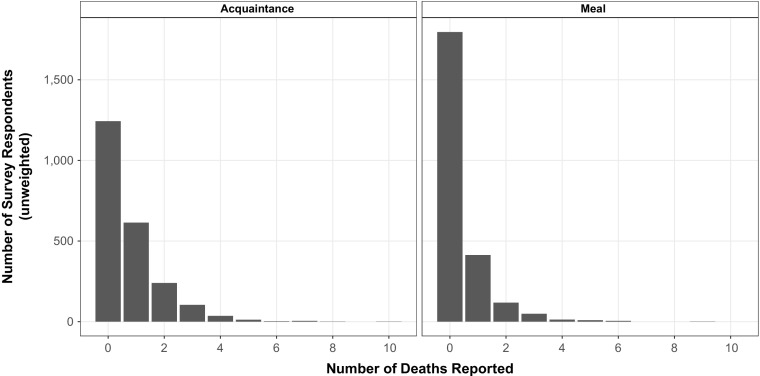
Distribution of the number of adult deaths reported by respondents using the acquaintance network (*left panel*) and the meal network (*right panel*)

To provide intuition about the information about deaths that the network reporting collects, we begin by reporting some basic descriptive statistics. Figure 2 shows the distribution of the number of deaths per interview in the two arms of the survey experiment. As expected, respondents reported knowing more deaths in the acquaintance condition (0.7 deaths per interview) than the meal condition (0.4 deaths reported per interview) (Table D4, Online Resource 1).

**Fig. 3:**
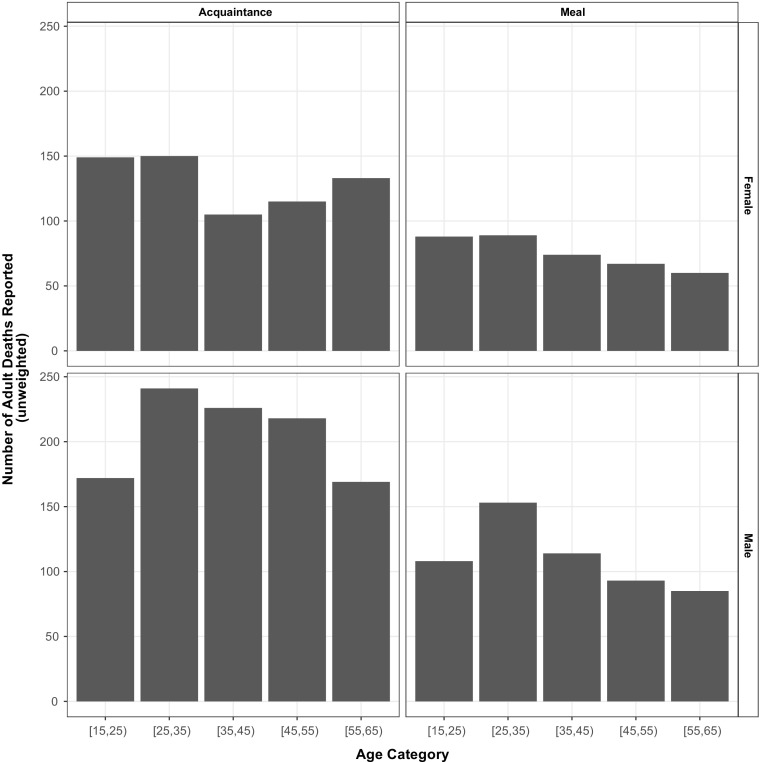
Age and sex distribution of adult deaths reported by respondents using the acquaintance network (*left panels*) and the meal network (*right panels*)

Figure 3 reports the age-sex distributions of the reported deaths in the two arms of the survey experiment.^3^ Online Resource 1 (section H) provides other descriptive plots, including those for (1) the responses for the groups of known size, (2) heaping in reported ages of death, and (3) a more detailed comparison between responses to the questions related to the network reporting method and sibling survival method.

### Network Survival Method Estimates

**Fig. 4:**
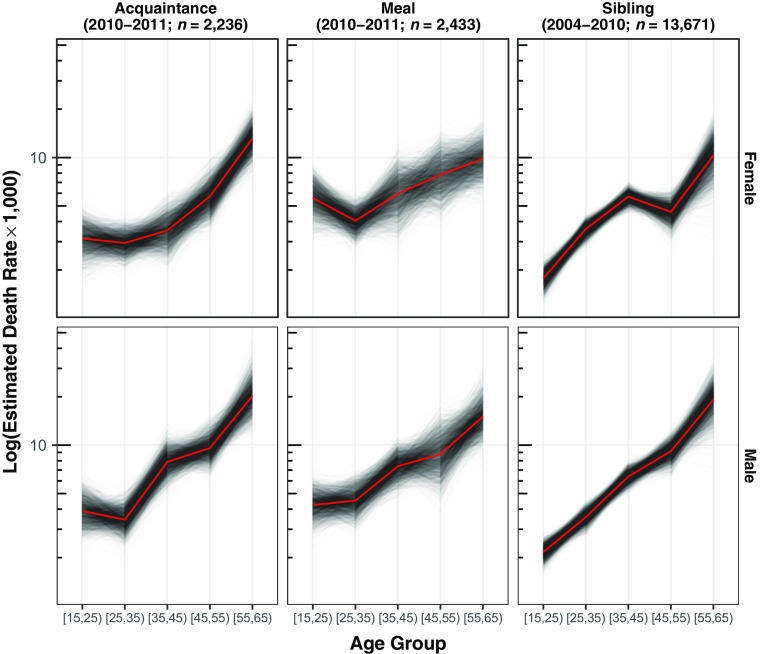
Comparison between network survival death rate estimates for two types of personal network (*left column and middle column*), and direct sibling survival death rates estimates from the 2010 Rwanda Demographic and Health Survey (*right column*). The top row has death rates estimated for females, and the bottom row has death rates estimated for males. The network survival estimates are based on reported deaths from the 12 months prior to the interview. The sibling estimates are based on reported deaths in the 84 months prior to the interview because estimates from the 12 months prior were too unstable (see Online Resource 1 (section F)). Each gray line shows the estimate from one bootstrap resample; taken together, the set of lines shows the estimated sampling uncertainty of the death rates. The thicker black lines show the mean of the bootstrap resamples

Figure 4 (left and middle columns) reports the estimated age-specific death rates (*M*_α_, Eq. (11)) across the two tie definitions for males and females.^4^ As expected, the estimated death rates generally increase with age (with the exception of young females for the meal definition).

**Fig. 5:**
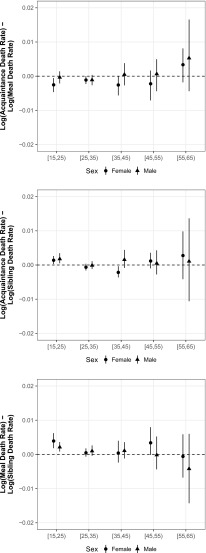
Age-specific differences between the estimated log death rate using (1) the acquaintance network and the meal network (*top panel*); (2) the acquaintance network and the sibling histories (*middle panel*); and (3) the meal network and the sibling histories (*bottom panel*). Above the dotted line, estimated death rates from the meal or acquaintance network are higher. These estimates are presented in tabular form in Online Resource 1 (section D)

The top panel of Fig. 5 directly plots the difference between estimates from the two tie definitions for different age groups, showing broad overall agreement between the estimates from each tie definition with the largest differences in the oldest age group. We discuss the middle and bottom panels of Fig. 5 in the upcoming section, Comparison With Estimates From the Sibling Survival Method.

## Comparison With Other Estimates

In addition to comparing our network survival estimates with each other, we also compare them with direct sibling survival estimates produced from the 2010 Rwanda DHS (NISR et al. 2012) and with estimates produced by three organizations: WHO, UNPD, and the Institute for Health Metrics and Evaluation (IHME). To foreshadow our results, we find that the network survival estimates were similar to the sibling survival estimates and to estimates from these three organizations.

### Comparison With Estimates From the Sibling Survival Method

The 2010 Rwanda DHS finished fieldwork in March 2011, right before our data collection started. As is typical in a DHS, only women of reproductive age (aged 15–49) were interviewed using the sibling survival module. Therefore, the sibling survival estimates we present are based on the sibling histories of the 13,671 women between ages 15 and 49 who were interviewed in the 12,540 households sampled in the DHS.

Even with 13,671 respondents, however, we found that estimated death rates for the 12 months before the survey were too imprecise to usefully compare with network survival estimates (Fig. F1, Online Resource 1). Therefore, we follow the recommendations of the sibling survival literature and pool together information from reports about 84 months (seven years) prior to the survey (Stanton et al. 2000; Timaeus and Jasseh 2004). The sibling survival estimates are thus estimated average death rates over the 84 months before the survey, whereas the network survival estimates are estimated death rates for the 12 months prior to the survey. (See Online Resource 1, section F, for detailed information about how we calculated sibling survival estimates.) As with the network survival estimates, we estimate the sampling uncertainty in the sibling survival estimates using the rescaled bootstrap, which accounts for the complex sample design of the DHS (Rao and Wu 1988; Rao et al. 1992).

Figure 4 shows the age-specific death rates produced from the network reporting method (left and middle columns) and the ones produced by the direct sibling survival method (right column). Further, Fig. 5 directly shows differences between the acquaintance and sibling estimates (middle panel) and between the meal and sibling estimates (bottom panel). This comparison shows that network survival estimates from both tie definitions are similar to the sibling survival estimates, even though the network survival estimates are based on a sample that is roughly one-fifth the size (*n* = 2*,*236 network reporting method (acquaintance); *n* = 2*,*433 network reporting method (meal); *n* = 13*,*671 sibling survival method). One systematic difference between the two methods is that the network survival estimates are slightly higher than sibling survival estimates for the youngest age group.

**Fig. 6:**
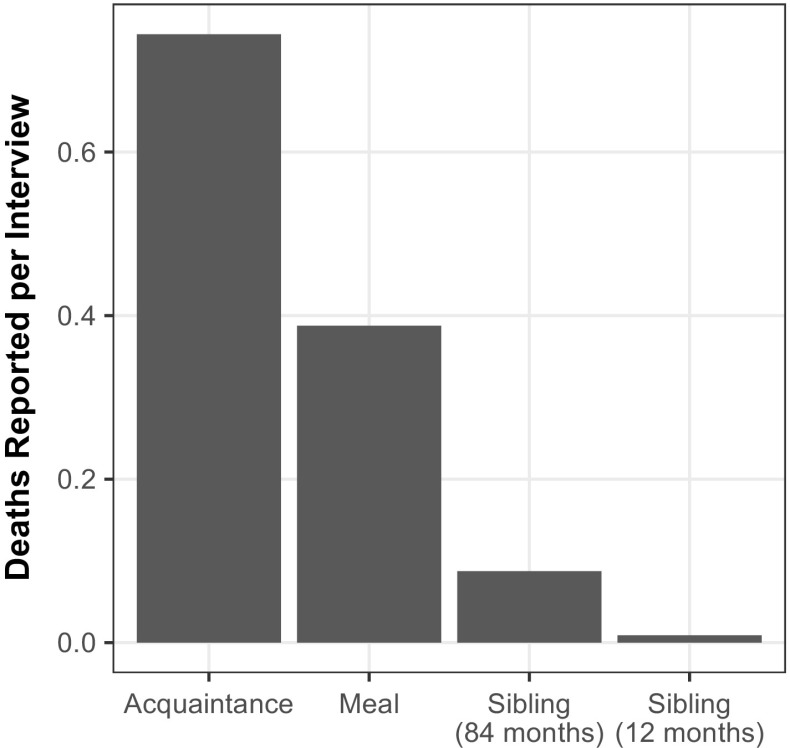
Average number of deaths reported from each interview in Rwanda using the acquaintance and meal tie definitions from the network survival study, and using the sibling history module of the DHS survey. The acquaintance and meal definitions use reported information about deaths in the 12 months prior to the survey. Compared with sibling reports about 84 months before the survey, network survival respondents reported approximately eight times more deaths using the acquaintance tie definition and approximately four times more deaths using the meal tie definition. Compared with the sibling reports about 12 months before the survey, network survival respondents reported approximately 82 times more deaths using the acquaintance tie definition and approximately 43 times more deaths using the meal tie definition

To clarify how the network survival method was able to produce similar estimates with substantially smaller samples, Fig. 6 compares the number of deaths reported per interview for the different approaches. Considering a 12-month reporting window, the network survival method yielded approximately 40 times (meal) or 80 times (acquaintance) more deaths per interview than the sibling survival method.^5^ Because it yields so many more deaths per interview than the sibling survival method, the network survival method can produce more granular estimates in samples of a similar size or can produce similar estimates with smaller samples.

### Comparison With Estimates From Organizations

In addition to comparing network survival estimates with sibling survival estimates, we also compare them with estimated adult mortality rates produced by three organizations: UNPD (United Nations Population Division 2015),^6^ WHO (WHO 2015),^7^ and the IHME (Nagavi et al. 2015).^8^

Researchers typically use estimates from these organizations to compare adult mortality across countries using an aggregate quantity called _45_ *q*_15_, which is the conditional probability of dying before age 60 among people who survive to age 15 and who then face the given age-specific death rates (Preston et al. 2001; Wachter 2014). For example, a set of age-specific death rates with _45_ *q*_15_ of 0.2 implies that 20 % of people who survive to age 15 and then face those age-specific death rates will die before age 60. The estimated _45_ *q*_15_ from each organization is derived from a complex combination of data sources, models, and expert judgment.^9^

**Fig. 7:**
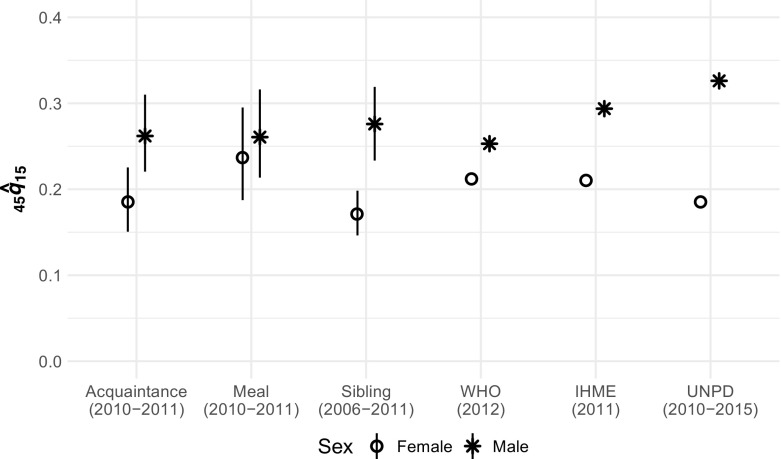
Estimated _45_ *q*_15_ for Rwanda from six sources: the acquaintance and meal tie definitions from our network survival method; the direct sibling survival method from the 2010 Rwanda Demographic and Health Survey; the United Nations Population Division (UNPD); the World Health Organization (WHO); and the Institute for Health Metrics and Evaluation (IHME). Error bars indicate 95 % sampling uncertainty intervals for the survey-based estimates, which were computed using the rescaled bootstrap. The estimates are not for exactly the same periods

Figure 7 compares estimated _45_ *q*_15_ for Rwanda from the network survival method with estimates from three organizations. (No sampling-based uncertainty estimates are available for the estimates from the organizations.) Figure 7 shows that estimates from the network survival method are similar to estimates from WHO and IHME, and to female estimates from UNPD (UNPD’s male _45_ *q*_15_ estimates are slightly higher than all of the other estimates). Figure 7 also shows that the difference between male and female mortality appears to be larger for the acquaintance network than for the meal network, a pattern that was not as apparent in Fig. 5. In Online Resource 1 (section F), we extend this comparison to age-specific death rates and again find that estimates from both arms of our survey experiment are similar to estimates from WHO, IHME, and UNPD (Fig. F2, Online Resource 1). The estimates from the network reporting method, however, did not require model life tables or other external data from neighboring countries or periods.

## Framework for Sensitivity Analysis

Any approach to estimating adult mortality rates will have to make assumptions. Unfortunately, it is not clear how the sibling survival method and the methods used by the organizations are affected by violations of their underlying assumptions. Because of the mathematical structure of the network survival method, however, we were able to derive a complete framework for sensitivity analysis. This framework shows analytically how the network survival estimates are affected by violations of assumptions, both individually and jointly.

We develop the full framework in Online Resource 1 (section C), which includes conditions related to (1) respondent reporting behavior, (2) social network structure, (3) questionnaire construction, and (4) sampling. Here, we illustrate the sensitivity framework by focusing on three important conditions, which were introduced earlier: the no false positives assumption, the decedent network condition, and the accurate reporting condition.

The network survival estimator’s sensitivity to these three important conditions is captured by the decomposition in Eq. (12), which relates the true number of deaths (*D*_α_) to the network survival estimand (${y}_{F,{D}_{\upalpha}}/{\overline{d}}_{F_{\upalpha},F}$) and three multiplicative adjustment factors (δ_*F*,α_, η_*F*,α_, and τ_*F*,α_):

12 $$ {D}_{\upalpha}=\underset{\begin{array}{l}\mathrm{network}\ \mathrm{survival}\\ {}\mathrm{estimand}\ \mathrm{f}\mathrm{o}\mathrm{r}{D}_{\alpha}\end{array}}{\underbrace{\left(\frac{y_{F,{D}_{\upalpha}}}{{\overline{d}}_{F_{\upalpha},F}}\right)}}\times \underset{\mathrm{adjustment}\ \mathrm{f}\mathrm{actors}}{\underbrace{\left(\frac{1}{\updelta_{F,\upalpha}}\right)\times \left(\frac{\upeta_{F,\upalpha}}{\uptau_{F,\upalpha}}\right)}}. $$

The first adjustment factor—the degree ratio (δ_*F*,α_)—is related to the structure of the underlying social network: it is exactly 1 when the decedent network assumption is satisfied, less than 1 if survey respondents in group α have bigger personal networks than people who died, and greater than 1 otherwise. The other two adjustment factors—the true positive rate (τ_*F*,α_) and the precision (η_*F*,α_)—are related to the accuracy of reporting; when respondents’ reports are perfectly accurate, then both τ_*F*,α_ and η_*F*,α_ are 1. If there are false positive reports, then the precision will be less than 1; if respondents do not report all deaths that actually happen in their personal networks, then the true positive rate will be less than 1. Online Resource 1 (section C) has more information, including precise definitions of each adjustment factor.

**Fig. 8:**
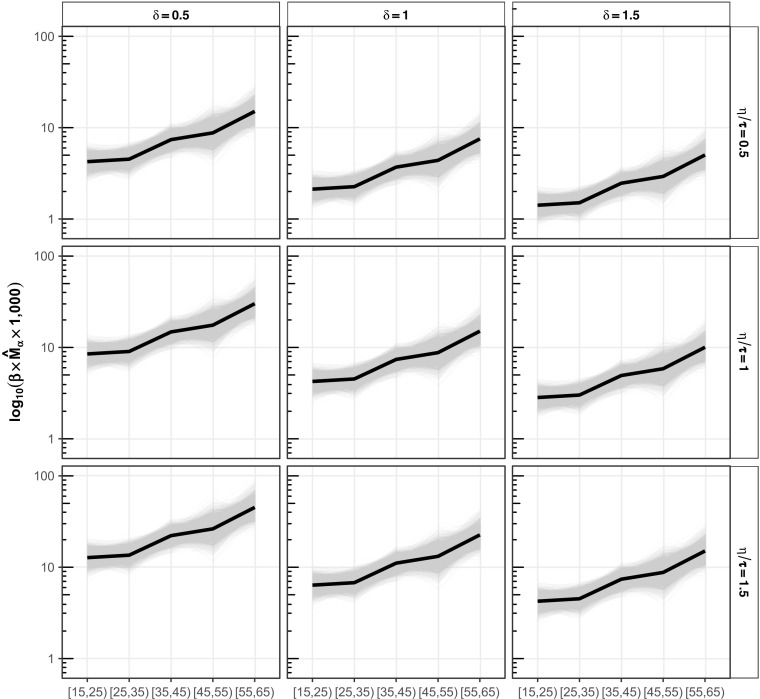
Estimated age-specific death rates for Rwandan males using the meal definition under violations of reporting and network structure conditions. The rows show different types of reporting: in the middle row, the accurate reporting condition holds (η_*F* , α_/τ_*F* , α_ = 1); in the top row, reporting tends to omit deaths (η_*F* , α_/τ_*F* , α_ = 0.5); and in the bottom row, reporting tends to erroneously include deaths (η_*F* , α_/τ_*F* , α_ = 1.5). The columns show different types of personal network structure: in the middle column, the decedent network condition holds (δ_*F* , α_ = 1); in the left column, people who die have smaller personal networks than the average frame population member (δ_*F* , α_ = 0:5); in the right column, people who die have personal networks that are larger than the average frame population member (δ_*F* , α_ = 1.5). Violations of the accurate reporting and decedent network condition can work in opposite directions, balancing each other out (top left and bottom right panels); or, they can work in the same direction, making estimates less accurate (bottom left and top right panels)

Figure 8 illustrates how the decomposition in Eq. (12) can be used to assess how death rate estimates are affected by (1) violations of the decedent network condition (δ_*F*,α_ = 1, columns), and (2) violations of the two reporting conditions (η_*F*,α_ */* τ_*F*,α_ = 1, rows). Fig. 8 shows that violations of these conditions can work in opposite directions, canceling each other’s effects (e.g., the bottom-right panel of Fig. 8); or they can work in the same direction, making the estimates less accurate (e.g., the bottom-left panel of Fig. 8). This example illustrates a small portion of the sensitivity framework in Online Resource 1 (section C), which can be used to assess how sensitive death rate estimates are to all the conditions required by the network survival estimator, individually and jointly.

## Discussion

Understanding adult mortality is critical to a wide range of important research and policy questions, but estimating adult death rates remains difficult in countries that lack high-quality vital registration systems. In this study, we introduced a promising new method for estimating adult death rates that overcomes many of the limitations of existing approaches, such as the sibling survival method. Our approach—the network survival method—uses information about survey respondents’ personal networks to estimate adult death rates.

In addition to deriving the theoretical properties of the network survival estimator and developing a framework for sensitivity analysis, we also designed and conducted a nationally representative survey experiment to test the method in Rwanda, a setting where improved methods for estimating adult mortality are sorely needed. We found that two versions of the network reporting method produced estimates that were similar to those produced by the sibling survival method, even though the network reporting estimates were based on a sample that was one-fifth the size. Further, the aggregated versions of the network survival estimates were comparable to the estimates from three organizations that incorporate data from multiple surveys and model life tables to create smoothed estimates.

Our results—theoretical and empirical—show that the network survival method can potentially overcome the two fundamental challenges in estimating death rates from surveys: it enables researchers to learn about people who died, and it can produce estimated death rates by age and sex from survey samples of moderate size.

The network survival method also has some potential advantages over the sibling survival method. First, the network survival method collects more information per interview than the sibling survival method. In our study in Rwanda, it collected approximately 80 times more reported deaths using the acquaintance tie definition and approximately 40 times more reported deaths using the meal tie definition (Fig. 6). By collecting more information per interview, the network reporting method was able to directly estimate adult death rates by age and sex for the 12 months prior to the survey without any pooling across countries or time. Because one of the main goals monitoring adult death rates is to detect—and react to—changes, the ability to produce direct, local, and timely estimates would be an improvement over current estimates that are pooled in a variety of different ways. Based on the high number of deaths reported per interview by network survival respondents in Rwanda, we believe that the network survival estimator could produce estimates of adult death rates for the past 12 months based only on data from a survey like the DHS.

Second, the network survival method has a formal framework for sensitivity analysis, which allows researchers to clearly identify and analytically quantify the effect of structural and reporting errors—and the interaction between them—on estimates. As a result, there is no ambiguity about how potential biases will affect network survival estimates, and it is straightforward to conduct routine sensitivity analyses of all estimates. Such a framework does not yet exist for the sibling survival method, which has been the subject of methodological uncertainty about different sources of bias and how they might interact.

There are many potential directions for future work. First, we believe that there should be additional studies assessing the quality of network survival estimates in countries without vital records systems and in countries where estimates can be compared with gold standard measures. Second, the flexibility of the network survival method means that the type of network respondents report about can be customized—and hopefully optimized—for different settings. For example, in one country, it might make sense to ask about the network of people who attend the same mosque; in a different country, it would make more sense to ask about people who attend the same church. This choice of tie definition has implications for the size and nature of reporting errors, structural biases, and sampling uncertainty. Therefore, future research should develop methods for choosing the optimal tie definition for each study. Third, although we focused on estimating national-level adult death rates as part of routine household surveys, there is a demand for survey-based approaches to estimate mortality in a wide range of other settings, including conflicts, natural disasters, famines, epidemic outbreaks, and other humanitarian crises (Checchi and Roberts 2008; Epicentre 2007). We believe that the network survival method could be tailored to work in some of these settings as well. Fourth, our survey interviewed adults of all ages, but some household surveys restrict the population that they interview by age or sex, potentially limiting the ability to produce reliable age-specific mortality rates for age groups other than those of the survey respondents (such as _60_ *q*_20_). Mortality among older age groups is becoming increasingly important to measure given the global shift toward monitoring mortality related to noncommunicable diseases that largely occur in the older age groups.^10^ We hope that the ideas in Online Resource 1 (section G) enable other researchers to modify our approach for these settings. Finally, we hope that the network survival method might help inspire improvements in the sibling survival method, particularly in terms of sensitivity analysis.

The scandal of invisibility means that almost two-thirds of deaths in the world are not recorded in a vital registration system (AbouZahr et al. 2015). The long-term solution is to develop effective vital registration systems in every country. Unfortunately, there has been very little progress improving the systems in developing countries over the past 15 years (Mikkelsen et al. 2015). Other demographic quantities, such as fertility and child mortality, were once as poorly understood as adult mortality is now. But today, even the world’s poorest countries have high-quality survey-based estimates of fertility and child mortality rates thanks to the development of appropriate survey-based methods and a massive, internationally coordinated infrastructure to deploy those methods around the world. The same infrastructure could also be harnessed to estimate adult mortality, and we believe that the network survival method is a promising step in that direction.

## Electronic supplementary material

Online Resource 1 (PDF 1251 kb)

## Footnotes

1 Another survey-based approach focuses on collecting information about deaths in the household (El Arifeen et al. 2014; Hill et al. 2006; Koenig et al. 2007).

2 To avoid complicating our notation, we use *D*_α_ to represent both the number of deaths and also the set of people who have died; the intended meaning should be clear from context.

3 Of the 3,853 reported deaths, 8 (0.2 %) were missing age, sex, or both. These reported deaths are excluded from this analysis.

4 All our estimates were computed in R (R Core Team 2014) using the following packages: *networkreporting* (Feehan and Salganik 2014), *surveybootstrap* (Feehan and Salganik 2016b), *plyr* (Wickham 2011), *dplyr* (Wickham and Francois 2015), *stringr* (Wickham 2012), *gg-plot2* (Wickham 2009), *devtools* (Wickham and Chang 2013), *stargazer* (Hlavac 2014), *car* (Fox and Weisberg 2011), and *gridExtra* (Auguie 2012). Also, following conventional practice in the network scale-up literature, all network reports about groups of known size were top-coded at 30, meaning that reported values greater than 30 were treated as 30; this top-coding affected 0.2 % of the responses.

5 Another way to compare the amount of information per interview is to compare the number of deaths reported with the network survival method (12-month reporting window) with the number of deaths reported with the sibling survival method (84-month reporting window). In this case, the network survival method yields four times (meal) or eight times (acquaintance) more deaths per interview than the sibling survival method.

6 UNPD estimates are taken from the 2015 revision of the World Population Prospects (http://esa.un.org/unpd/wpp/Download/Standard/ASCII/).

7 WHO estimates are taken from the Global Health Observatory (http://www.who.int/gho/database/en/ and http://apps.who.int/gho/data/view.main.61370).

8 IHME estimates are taken from the 2013 Global Burden of Disease study (http://ghdx.healthdata.org/global-burden-disease-study-2013-gbd-2013-data-downloads).

9 In brief, the methods used to estimate adult mortality for WHO and the UNPD are fairly similar: data from censuses and household surveys (such as the DHS) are combined with model life tables to estimate the adult mortality levels. These estimates, therefore, rely on extrapolating adult mortality from estimates of child mortality levels (see Masquelier et al. 2014, for a more detailed discussion). For IHME, a smoothed regression approach is taken that incorporates additional variables related to health and borrows strength from data from other countries and periods. For more information about how these organizations produce estimates, see United Nations Population Division (2015), Wang et al. (2013), and WHO (2015).

10 See Target 3.4 (http://unstats.un.org/sdgs/metadata).
