# Supplementary material for: The Network Survival Method for Estimating Adult Mortality: Evidence From a Survey Experiment in Rwanda
Source: Demography. 2017 Jul 24;54(4):1503–28. doi: 10.1007/s13524-017-0594-y (PMC5547188; doi:10.1007/s13524-017-0594-y)
Supplement: Supplementary file 1 — (PDF 1251 kb) [file 13524_2017_594_MOESM1_ESM.pdf]

Online Resource 1

The Network Survival Method for Estimating Adult Mortality: Evidence From a Survey Experiment in Rwanda

Dennis M. Feehan, Mary Mahy, and Matthew J. Salganik

A Estimating personal network size

The network survival estimator uses the personal networks of survey respondents in demographic group α to estimate the visibility of deaths in demographic group α. This approach requires a method for estimating the average personal network size of survey respondents in demographic group α, $\bar{d}_{F_\alpha,F}$. In this appendix, we adapt an existing personal network size estimator called the known population method (Killworth et al., 1998a) so that it can be used to estimate $\bar{d}_{F_\alpha,F}$. Most of the contents of this appendix closely parallel the formal analysis of the known population estimator in Feehan and Salganik (2016a, Online Appendix B).

Before presenting the first result, we first need to introduce some notation for working with the groups of known size. Let U be the entire population (e.g., all of Rwanda), and let F be the frame population for the survey (e.g., Rwandan adults). Suppose that we have several groups $A_1, A_2, \ldots, A_J$ with $A_J \subset U$. These groups are the known populations. Imagine concatenating all of the people in populations $A_1, A_2, \ldots, A_J$ together, repeating each individual once for each population she is in. The result, which we call the *probe alters* $\mathcal{A}$ is a multiset. The size of $\mathcal{A}$ is $N_{\mathcal{A}} = \sum_j N_{A_j}$. In our notation, we use $\mathcal{A}$ in subscripts like any other set; for example, $y_{F_\alpha,\mathcal{A}}$ is the reported connections from frame population members in group α (F_α) to the probe alters ($\mathcal{A}$).

Result A.1 *Suppose we have a probability sample s taken from the frame population with known probabilities of inclusion π_i. Further, suppose we have a multiset of probe alters $\mathcal{A}$ that have been chosen so that two conditions hold:*

- $y_{F_\alpha,\mathcal{A}} = d_{F_\alpha,\mathcal{A}}$ *(reporting condition)*
- $\bar{d}_{\mathcal{A},F_\alpha} = \bar{d}_{F,F_\alpha}$ *(probe alter condition).*

Then the adapted known population estimator

$$\widehat{\bar{d}}_{F_\alpha,F} = \frac{\sum_{i \in s_\alpha} y_{i,\mathcal{A}}/\pi_i}{\sum_j N_{A_j}} \; \frac{N_F}{N_{F_\alpha}} \tag{A.1}$$

is consistent and unbiased for $\bar{d}_{F_\alpha,F}$.

Proof: By Property B.2 of Feehan and Salganik (2016a), $\widehat{y}_{F_\alpha,\mathcal{A}}/N_\mathcal{A}$ is consistent and unbiased for $y_{F_\alpha,\mathcal{A}}/N_\mathcal{A}$. By the reporting condition, $y_{F_\alpha,\mathcal{A}}/N_\mathcal{A} = d_{F_\alpha,\mathcal{A}}/N_\mathcal{A}$. Re-writing this quantity, we have

$$\frac{d_{F_\alpha,\mathcal{A}}}{N_\mathcal{A}} = \frac{d_{\mathcal{A},F_\alpha}}{N_\mathcal{A}} = \bar{d}_{\mathcal{A},F_\alpha}. \tag{A.2}$$

Now, using the probe alter condition,

$$\bar{d}_{\mathcal{A},F_\alpha} = \bar{d}_{F,F_\alpha}. \tag{A.3}$$

So we have shown that, assuming the reporting condition and the probe alter condition hold, $\widehat{y}_{F_\alpha,\mathcal{A}}/N_\mathcal{A}$ is consistent and unbiased for $\bar{d}_{F,F_\alpha}$. Now we can re-write $\bar{d}_{F,F_\alpha}$ as

$$\bar{d}_{F,F_\alpha} = \frac{d_{F,F_\alpha}}{N_F} = \frac{d_{F_\alpha,F}}{N_F}. \tag{A.4}$$

So we conclude that the estimator is consistent and unbiased for

$$\frac{d_{F_\alpha,F}}{N_F}\,\frac{N_F}{N_{F_\alpha}} = \frac{d_{F_\alpha,F}}{N_{F_\alpha}} = \bar{d}_{F_\alpha,F}. \tag{A.5}$$

■

Feehan and Salganik (2016a, Online Appendix B) offers suggestions for how to choose probe alters for the known population estimator; these suggestions carry over to the adapted estimator (Result A.1) with some modifications to accommodate the specific reporting condition and probe alter condition required by the adapted known population estimator.

B The network survival estimator

In this appendix, we provide formal results related to the network survival estimator. Several of the results in this appendix follow the analysis of the generalized scale-up estimator found in Feehan and Salganik (2016a).

B.1 Estimating the number of deaths, D_α

Equation 5 shows that the two components of the estimated number of deaths are: (i) the total number of reports about deaths, y_{F,D_α}; and (ii) the average visibility of deaths, $\bar{v}_{D_\alpha,F}$. First, we present results about estimators for each of these two components. Then we show that estimators for these two components can be combined to estimate M_α.

Result B.1, shows that y_{F,D_α} can be estimated from survey reports using standard survey techniques.

Result B.1 *Suppose we have a probability sample s taken from the frame population with known probabilities of inclusion π_i. Then*

$$\widehat{y}_{F,D_\alpha} = \sum_{i \in s} y_{i,D_\alpha}/\pi_i \tag{B.1}$$

is consistent and unbiased for y_{F,D_α}.

Proof: Equation B.1 is a standard Horvitz-Thompson estimator (see, eg Sarndal et al., 2003, chap. 2), so it is consistent and unbiased for the total $\sum_{i \in F} y_{i,D_\alpha} = y_{F,D_\alpha}$. ■

Next, Result B.2 shows that it is possible to use information about survey respondents' personal networks to estimate the visibility of deaths ($\bar{v}_{F,D_\alpha}$) if two additional conditions are satisfied: the visible deaths condition and the decedent network condition.

Result B.2 *Suppose that* $\widehat{\bar{d}}_{F_\alpha,F}$ *is a consistent and unbiased estimator for* $\bar{d}_{F_\alpha,F}$ *(such as the one in Result A.1). Furthermore, suppose that the following conditions hold:*

- $\bar{v}_{D_\alpha,F} = \bar{d}_{D_\alpha,F}$ *(visible deaths condition)*
- $\bar{d}_{D_\alpha,F} = \bar{d}_{F_\alpha,F}$ *(decedent network condition)*

Then $\widehat{\bar{d}}_{F_\alpha,F}$ *is a consistent and unbiased estimator for* $\bar{v}_{D_\alpha,F}$.

Proof: By assumption, $\widehat{\bar{d}}_{F_\alpha,F}$ is consistent and unbiased for $\bar{d}_{F_\alpha,F}$. By the decedent network condition, $\bar{d}_{F_\alpha,F} = \bar{d}_{D_\alpha,F}$. And, by the visible deaths condition, $\bar{d}_{D_\alpha,F} = \bar{v}_{D_\alpha,F}$. ■

The *visible deaths condition* says that the average number of times a death could be reported (the visibility of deaths) is the same as the average number of network connections people who died have to the frame population (i.e., $\bar{v}_{D_\alpha,F} = \bar{d}_{D_\alpha,F}$). Substantively, we would expect this condition to hold when, on average, people who are connected to a person who died are aware of that fact and report it on a survey.

The *decedent network condition* says that the average size of personal networks is the same for dead people and for the people who respond to the survey (i.e., $\bar{d}_{D_\alpha,F} = \bar{d}_{F_\alpha,F}$). For example, suppose that women aged 50-54 who are eligible to be sampled by our survey have an average personal network size of 100. In that case, the decedent network condition is satisfied when women aged 50-54 who died also have an average personal network size of 100.

The visible death condition and the decedent network condition could both be violated in practice. Therefore, in Online Appendix C we develop a sensitivity analysis framework that enables researchers to understand the impact that violations of these two assumptions will have on the accuracy of estimated death rates.

Next, Result B.3 shows how the network survival method combines Results B.1 and B.2 to form an estimator for the number of deaths (D_α).

Result B.3 *Suppose* $\widehat{y}_{F,D_\alpha}$ *is a consistent and unbiased estimator for* y_{F,D_α}*, and that* $\widehat{\bar{v}}_{D_\alpha,F}$ *is a consistent and unbiased estimator for* $\bar{v}_{D_\alpha,F}$*. Suppose also that there are no false positive reports, so that* $v_{i,F} = 0$ *for all* $i \notin D_\alpha$*. Then*

$$\widehat{D}_\alpha = \frac{\widehat{y}_{F,D_\alpha}}{\widehat{\bar{v}}_{D_\alpha,F}} \tag{B.2}$$

is consistent and essentially unbiased for D_α*.*

Proof: With consistent and unbiased estimators for y_{F,D_α} and for $\bar{v}_{D_\alpha,F}$, we can form a consistent and essentially unbiased estimator for $y_{F,D_\alpha}/\bar{v}_{D_\alpha,F}$ using a standard ratio approach (Sarndal et al., 2003, chap. 5)[11]. So it remains to show that $y_{F,D_\alpha}/\bar{v}_{D_\alpha,F} = D_\alpha$. Since in-reports must equal out-reports (see Feehan (2015) and Feehan and Salganik (2016a)), $y_{F,D_\alpha} = v_{U,F}$, where U is the set of all of the people who could be reported about, living or dead (note that $D_\alpha \subset U$ and $F \subset U$). By the no false positives assumption, $v_{i,F} = 0$ for all $i \notin D_\alpha$, which means that

$$v_{U,F} = \sum_{i \in U} v_{i,F} = \sum_{i \in D_\alpha} v_{i,F} = v_{D_\alpha,F}. \tag{B.3}$$

So we conclude that $y_{F,D_\alpha} = v_{D_\alpha,F}$. Dividing both sides of this identity by D_α and re-arranging produces

$$D_\alpha = \frac{y_{F,D_\alpha}}{v_{D_\alpha,F}/D_\alpha} = \frac{y_{F,D_\alpha}}{\bar{v}_{D_\alpha,F}}. \tag{B.4}$$

■

[11] Ratio estimator are standard in survey research, and a discussion of them can be found in many texts. Ratio estimators are not, strictly speaking, unbiased. However, there is a large literature that confirms that the bias in ratio estimators is typically very small when samples are not too small (see, for example, Sarndal et al. (2003, chap. 5); Feehan and Salganik (2016a, Online Appendix E); and Rao and Pereira (1968)). Since ratio estimators are technically biased, but the bias can be expected to be very small, we use by the term *essentially unbiased* instead of unbiased in several of our results.

B.2 Estimator for M_α

We now turn to a set of results related to estimating the death rate M_α[12]. We begin by developing a general expression that can be used to estimate the death rate M_α. Then we discuss, in detail, the way that we used the general expression to estimate death rates in our study.

We begin with a general result.

Result B.4 *Suppose we have a probability sample s taken from the frame population with known probabilities of inclusion π_i. Suppose also that we have a consistent and unbiased estimator $\widehat{y}_{F,D_\alpha}$ (eg, Result B.1); a consistent and unbiased estimator $\widehat{\bar{v}}_{D_\alpha,F}$ (eg, Result B.2); and a consistent and unbiased estimator $\widehat{N}_\alpha$. Then*

$$\widehat{M}_\alpha = \frac{\widehat{y}_{F,D_\alpha}}{\widehat{\bar{v}}_{D_\alpha,F}} \frac{1}{\widehat{N}_\alpha} \tag{B.5}$$

is consistent and essentially unbiased for $M_\alpha = D_\alpha / N_\alpha$.

Proof: Equation B.5 is a compound ratio estimator; Rao and Pereira (1968) and Feehan and Salganik (2016a, Online Appendix E) give proofs that compound ratio estimators are consistent and essentially unbiased. ■

Result B.4 is very general in the sense that it can be used to estimate death rates by combining any consistent and unbiased estimators of connections to people who died, the visibility of deaths, and the size of the population. For our study, we customized this general estimator in two ways. First, we used the adapted known population estimator for $\bar{d}_{F_\alpha,F}$ (Result A.1) as an estimator of the visibility of deaths ($\bar{v}_{D_\alpha,F}$). Second, we assumed that the sampling frame was complete ($N_{F_\alpha} = N_\alpha$ for all α)[13]

[12]Note that, as is typical in demographic research, we use the size of the population to approximate the exposure in the denominator of the death rate. This approximation should not be problematic unless (i) the time period over which death rates are computed is long; or (ii) death rates are extremely high (much higher than populations typically experience). For the 12-month death rates we study in Rwanda, we do not expect this approximation to pose a problem.

[13]In our study, we believe that it is reasonable to assume that the sampling frame was complete

These two choices lead to a more specific estimator that we used in this study.

Result B.5 *Suppose we have a probability sample s taken from the frame population with known probabilities of inclusion π_i. Suppose that we have a set of probe alters $\mathcal{A}$ (also called known populations) that satisfy the reporting condition ($y_{F_\alpha,\mathcal{A}} = d_{F_\alpha,\mathcal{A}}$) and the probe alter condition ($\bar{d}_{\mathcal{A},F_\alpha} = \bar{d}_{F,F_\alpha}$) from Result A.1. Suppose that the visible deaths condition ($\bar{v}_{D_\alpha,F} = \bar{d}_{D_\alpha,F}$) and the decedent network condition ($\bar{d}_{D_\alpha,F} = \bar{d}_{F_\alpha,F}$) from Result B.2 are satisfied. Finally, suppose that the frame population is complete, ($N_{F_\alpha} = N_\alpha$), and that there are no false positive reports about deaths ($v_{i,F} = 0$ for all $i \notin D_\alpha$). Then*

$$\widehat{M}_\alpha = \frac{\sum_{i\in s} y_{i,D_\alpha}/\pi_i}{\sum_{i\in s_\alpha} y_{i,\mathcal{A}}/\pi_i} \frac{N_\mathcal{A}}{N_F} = \frac{\widehat{y}_{F,D_\alpha}}{\widehat{y}_{F_\alpha,\mathcal{A}}} \frac{N_\mathcal{A}}{N_F} = \frac{\widehat{y}_{F,D_\alpha}}{\widehat{\bar{d}}_{F_\alpha,F} \times N_{F_\alpha}} \tag{B.6}$$

is consistent and essentially unbiased for $M_\alpha = D_\alpha/N_\alpha$.

Proof: First, note that

$$\frac{\widehat{y}_{F,D_\mathcal{A}}}{\widehat{\bar{d}}_{F_\alpha,F} \times N_{F_\alpha}} = \frac{\widehat{y}_{F,D_\alpha}}{\widehat{y}_{F_\alpha,\mathcal{A}}} \frac{N_\mathcal{A}}{N_{F_\alpha}} \frac{N_{F_\alpha}}{N_F} \tag{B.7}$$

$$= \frac{\widehat{y}_{F,D_\alpha}}{\widehat{y}_{F_\alpha,\mathcal{A}}} \frac{N_\mathcal{A}}{N_F}, \tag{B.8}$$

where we have plugged in the definition of the adapted known population estimator and cancelled the N_{F_α} (Result A.1).

Equation B.8 is a standard ratio estimator, so it is consistent and essentially unbiased

(i.e., that all adults could have been selected) because of our field procedures. More specifically, our approach was to (1) randomly sample a set of geographical areas; (2) send a team to visit the geographical areas and produce a census of dwellings; and then (3) choose a sample of dwellings and interview all adults who lived in them. See Rwanda Biomedical Center/Institute of HIV/AIDS et al. (2012) for more information about the sampling design. Researchers concerned about either of these choices can use the sensitivity framework in Online Appendix C to assess the sensitivity of the estimated death rates to this assumption.

for the quantity

$$Q_\alpha = \frac{y_{F,D_\alpha}}{y_{F_\alpha,\mathcal{A}}} \frac{N_\mathcal{A}}{N_F} \tag{B.9}$$

(see, e.g. Sarndal et al., 2003, chap. 5). So it remains to show that $Q_\alpha = D_\alpha/N_\alpha = M_\alpha$. We will do this by working backwards through the discussion above. First, multiply Q_α by N_{F_α}/N_α (which equals 1, by the completeness of the frame population), to obtain

$$Q_\alpha = \frac{y_{F,D_\alpha}}{y_{F_\alpha,\mathcal{A}}} \frac{N_\mathcal{A}}{N_F} \frac{N_{F_\alpha}}{N_\alpha}. \tag{B.10}$$

Now we can use the reporting condition ($y_{F_\alpha,\mathcal{A}} = d_{F_\alpha,\mathcal{A}}$) followed by the probe alter condition ($\bar{d}_{\mathcal{A},F_\alpha} = \bar{d}_{F,F_\alpha}$) to rewrite the expression as

$$Q_\alpha = \frac{y_{F,D_\alpha}}{\bar{d}_{F,F_\alpha}} \frac{1}{N_F} \frac{N_{F_\alpha}}{N_\alpha}. \tag{B.11}$$

Now, recall that $\bar{d}_{F,F_\alpha}\ N_F/N_{F_\alpha} = \bar{d}_{F_\alpha,F}$. Applying this relationship to simplify the denominator of Eq. B.11 produces

$$Q_\alpha = \frac{y_{F,D_\alpha}}{\bar{d}_{F_\alpha,F}} \frac{1}{N_\alpha}. \tag{B.12}$$

Finally, applying the decedent network condition ($\bar{d}_{F_\alpha,F} = \bar{d}_{D_\alpha,F}$) and the visible deaths condition ($\bar{d}_{D_\alpha,F} = \bar{v}_{D_\alpha,F}$), we have

$$Q_\alpha = \frac{y_{F,D_\alpha}}{\bar{v}_{D_\alpha,F}} \frac{1}{N_\alpha}. \tag{B.13}$$

Now, since there are no false positive reports, we can apply the argument in Result B.3 to conclude that $y_{F,D_\alpha}/\bar{v}_{D_\alpha,F} = D_\alpha$. Therefore,

$$Q_\alpha = \frac{D_\alpha}{N_\alpha} = M_\alpha. \tag{B.14}$$

■

C Sensitivity framework

The network survival estimator we used in Rwanda relies on several conditions (Result B.5), and these conditions can be separated into four groups: (i) reporting (for example, the visible deaths condition); (ii) network structure (the decedent network connection); (iii) survey construction (for example, choosing the probe alters for the adapted known population method); and (iv) sampling (the requirement that researchers obtain a probability sample). In practice, we expect that researchers may not be sure that all of the conditions required by the network survival estimator are exactly satisfied. Therefore, in this appendix we develop a framework that researchers can use to quantitatively assess how violating each condition impacts estimated death rates. Our framework also identifies precise and well-defined quantities that future studies may be able to measure. With measurements for these quantities, network survival estimates could be adjusted and potentially improved[14].

In the next section, we focus on the impact of nonsampling errors. Then, we turn to an analysis of the impact of sampling errors. Finally, we combine the results into a unified sensitivity framework for network survival estimates.

C.1 Network survival sensitivity to nonsampling errors

To understand how different sources of nonsampling error affect network survival estimates, we will briefly review the network reporting framework; see Feehan (2015) and Feehan and Salganik (2016a) for more detail. Figure 1(b) shows an example

[14]Note that this framework is an adapted version of the one introduced for the scale-up estimator in Feehan and Salganik (2016a), and rigorous proofs for our sensitivity results can be found there. Moreover, to keep our derivations as simple as possible, our focus here will be on the specific estimator we used in Rwanda (Result B.5); however, by following the approach in this appendix, researchers can extend our approach to the more general estimator in Result B.4 as well.

of a reporting network that has been rearranged into a *bipartite reporting graph.* The edges in this bipartite reporting graph represent the reports that people in the frame population make about people who died. The edges contribute two types of quantities to the vertices in the graph: each edge adds an *out-report* to the people who do the reporting (F, on the left-hand side of the graph); and each edge also adds an *in-report* to the people who get reported about (U, on the right-hand side of the graph). We call the sum of all of the out-reports y_{F,D_α}, and the sum of all of the in-reports $v_{U,F}$.

Out-reports can be separated into two groups: (i), *true positives*, which are reports that correctly lead to people who died; and (ii) *false positives*, which are reports that incorrectly lead to people who did not die. We write the true positives as y^+_{F,D_α}, and the false positives as y^-_{F,D_α}. By definition, all of the true positive reports lead to D_α, meaning that $y^+_{F,D_\alpha} = v_{D_\alpha,F}$. This identity is true in *any* bipartite reporting graph, no matter how accurate or inaccurate respondents' reports are. Starting from $y^+_{F,D_\alpha} = v_{D_\alpha,F}$, multiplying both sides by D_α, and then rearranging the terms yields an identity that is the basis for the network survival estimator:

$$D_\alpha = \frac{y^+_{F,D_\alpha}}{\bar{v}_{D_\alpha,F}}. \tag{C.1}$$

Now we will use the network reporting framework to develop an expression for the sensitivity of network survival estimates for M_α, the death rate. Our approach will be to introduce quantities that capture the extent to which each required condition is satisfied. We call these quantities *adjustment factors.*

First, we focus on an expression for the sensitivity of the estimator for D_α, the number of deaths. Estimating the number of deaths requires that three conditions are satisfied: two reporting conditions and one condition related to network structure. The first condition required to estimate the number of deaths is that there are no false positive reports. To account for this requirement, we introduce a quantity called

the *precision*:

$$\eta_{F,\alpha} = \frac{\text{total \# of out-reports from frame popn that correctly lead to deaths}}{\text{total \# of out-reports from frame popn}} = \frac{y^{+}_{F,D_\alpha}}{y_{F,D_\alpha}}. \tag{C.2}$$

$\eta_{F,\alpha}$ relates accurate network reports to all network reports; it will range from 1, when reporting is perfectly accurate, to 0, when none of the out-reports correctly leads to a death. Values of $\eta_{F,\alpha}$ other than 1 mean that the no false positives assumption is violated.

The second condition required to estimate the number of deaths is the visible deaths condition. To account for this requirement, we introduce a quantity called the *true positive rate*:

$$\tau_{F,\alpha} = \frac{\text{avg \# of in-reports from the frame to each death}}{\text{avg \# of network connections from a death to the frame population}} = \frac{\bar{v}_{D_\alpha,F}}{\bar{d}_{D_\alpha,F}}. \tag{C.3}$$

$\tau_{F,\alpha}$ relates network degree to network reports; it will range from 1, when reporting is perfectly accurate, to 0, when no network edges leading to deaths are reported. Values of $\tau_{F,\alpha}$ other than 1 mean that the visible deaths condition is violated.

The third condition required to estimate the number of deaths is the decedent network condition. To account for this requirement, we introduce a quantity called the *degree ratio*:

$$\delta_{F,\alpha} = \frac{\text{avg \# edges from a death in } \alpha \text{ to the frame population}}{\text{avg \# edges from a frame pop member in } \alpha \text{ to the entire frame pop}} = \frac{\bar{d}_{D_\alpha,F}}{\bar{d}_{F_\alpha,F}}. \tag{C.4}$$

$\delta_{F,\alpha}$ will range from 0 to infinity. When it is less than one, people who die in demographic group α tend to have fewer connections to the frame population than frame population members in demographic group α; when it is greater than one, people who die in demographic group α tend to have more connections to the frame

population than frame population members in demographic group α. Values of $\delta_{F,\alpha}$ other than 1 mean that the decedent network condition is violated.

Together, the adjustment factors can be used to propose a decomposition of the difference between network survival estimand for D_α and the true value of D_α:

$$D_\alpha = \underbrace{\left(\frac{y_{F,D_\alpha}}{\bar{d}_{F_\alpha,F}}\right)}_{\substack{\text{network}\\\text{survival}\\\text{estimand}}} \times \underbrace{\underbrace{\frac{1}{\bar{d}_{D_\alpha,F}/\bar{d}_{F_\alpha,F}}}_{\substack{\text{degree ratio}\\\delta_{F,\alpha}}} \times \underbrace{\frac{1}{\bar{v}_{D_\alpha,F}/\bar{d}_{D_\alpha,F}}}_{\substack{\text{true positive rate}\\\tau_{F,\alpha}}} \times \underbrace{\frac{y^{+}_{F,D_\alpha}}{y_{F,D_\alpha}}}_{\substack{\text{precision}\\\eta_{F,\alpha}}}}_{\text{adjustment factors}}. \tag{C.5}$$

The decomposition in Eq. C.5, shows that the network survival estimand will estimate the true number of deaths if the three adjustment factors satisfy $\eta_{F,\alpha}/(\delta_{F,\alpha} \times \tau_{F,\alpha}) = 1$.

C.1.1 Sensitivity of the adapted known population estimator

We now analyze the sensitivity of the adapted known population estimator (Result A.1) to nonsampling conditions. The adapted known population estimator is used to estimate the size of survey respondents' personal networks; it requires three nonsampling conditions: first, that researchers have accurate information about the size of the known populations ($N_{\mathcal{A}}$); second, the probe alter condition ($\bar{d}_{\mathcal{A},F_\alpha} = \bar{d}_{F,F_\alpha}$); and third, the reporting condition ($y_{F_\alpha,\mathcal{A}} = d_{F_\alpha,\mathcal{A}}$). Following the strategy above, we introduce a quantitative adjustment factor to capture the extent to which each of these three conditions is satisfied. For example, suppose that in a particular study, the reporting condition is not satisfied, so that $y_{F_\alpha,\mathcal{A}} \neq d_{F_\alpha,\mathcal{A}}$; in that case, we can write $y_{F_\alpha,\mathcal{A}} = c d_{F_\alpha,\mathcal{A}}$ for some constant c; when $c = 1$, the condition is satisfied. The corresponding adjustment factor is then $c = \frac{y_{F_\alpha,\mathcal{A}}}{d_{F_\alpha,\mathcal{A}}}$.

By introducing an adjustment factor for each of the three assumptions— $c_1 = \frac{\widehat{N}_{\mathcal{A}}}{N_{\mathcal{A}}}$

for the known population totals, $c_2 = \frac{\bar{d}_{\mathcal{A},F_\alpha}}{\bar{d}_{F,F_\alpha}}$ for the probe alter condition, and $c_3 = \frac{y_{F_\alpha,\mathcal{A}}}{d_{F_\alpha,\mathcal{A}}}$ for the reporting conditions—the adapted known population estimator can be decomposed as:

$$\bar{d}_{F_\alpha,F} = \underbrace{\left(\frac{\widehat{y}_{F_\alpha,\mathcal{A}}}{\widehat{N}_{\mathcal{A}}} \frac{N_F}{N_{F_\alpha}}\right)}_{\substack{\text{adapted} \\ \text{known population}}} \times \underbrace{c_1}_{\substack{\text{known} \\ \text{population} \\ \text{totals}}} \times \underbrace{\frac{1}{c_2}}_{\substack{\text{probe} \\ \text{alter} \\ \text{condition}}} \times \underbrace{\frac{1}{c_3}}_{\substack{\text{reporting} \\ \text{conditions} \\ \text{for known} \\ \text{populations}}} . \tag{C.6}$$

C.1.2 Sensitivity to nonsampling conditions

We have now developed expressions that illustrate the sensitivity of estimands for y_{F,D_α}, D_α, and $\bar{d}_{F_\alpha,F}$. The final condition required by the estimator we used in Rwanda (Result B.5) is that the frame population be complete, meaning that $N_{F_\alpha} = N_\alpha$. Following the approach in the previous sections, we account for this condition by introducing the adjustment factor $c_4 = \frac{N_{F_\alpha}}{N_\alpha}$. With this final adjustment factor, we can combine our analysis of all of the nonsampling factors to produce

$$M_\alpha = \underbrace{\left(\frac{y_{F,D_\alpha}}{y_{F_\alpha,\mathcal{A}}} \times N_{\mathcal{A}}\right)}_{\substack{\text{network} \\ \text{survival} \\ \text{estimand} \\ \text{(Result B.5)}}} \times \underbrace{\frac{c_2\ c_3}{c_1}}_{\substack{\text{adapted} \\ \text{known} \\ \text{population} \\ \text{conditions}}} \times \underbrace{\frac{1}{c_4}}_{\substack{\text{frame} \\ \text{population} \\ \text{is complete}}} \times \underbrace{\frac{\eta_{F,\alpha}}{\tau_{F,\alpha}\delta_{F,\alpha}}}_{\substack{\text{reporting and} \\ \text{network} \\ \text{structure}}}. \tag{C.7}$$

To assess the sensitivity of death rate estimates to any of the nonsampling conditions required by network survival, researchers can (1) assume values for c_1, c_2, c_3, c_4, $\eta_{F,\alpha}$, $\tau_{F,\alpha}$, and $\delta_{F,\alpha}$ that describe how the conditions are not satisfied; and (2) plug these values into Equation C.7 to obtain the resulting death rate.

C.2 Sensitivity to sampling conditions

The last type of condition required by the network survival estimator is that researchers have obtained a probability sample and the associated sampling weights.

We begin by repeating Feehan and Salganik (2016a)'s definition of *imperfect sampling weights*, since this concept is critical to understanding the network survival estimator's sensitivity to sampling error.

Imperfect sampling weights. Suppose a researcher obtains a probability sample s_F from the frame population F (Sarndal et al., 2003). Let I_i be the random variable that assumes the value 1 when unit $i \in F$ is included in the sample s_F, and 0 otherwise. Let $\pi_i = \mathbb{E}[I_i]$ be the true probability of inclusion for unit $i \in F$, and let $w_i = \frac{1}{\pi_i}$ be the corresponding design weight for unit i. We say that researchers have *imperfect sampling weights* when researchers use imperfect estimates of the inclusion probabilities π'_i and the corresponding design weights $w'_i = \frac{1}{\pi'_i}$. Note that we assume that both the true and the imperfect weights satisfy $\pi_i > 0$ and $\pi'_i > 0$ for all i.

Feehan and Salganik (2016a, Result D.10) introduces two more quantities that we will use here. The first quantity, called ϵ_i, captures the relative error in the imperfect sampling weights for each unit i in the frame population. It is defined as $\epsilon_i = \frac{\pi_i}{\pi'_i}$. The second quantity is an index, called K, that depends on the quantity being estimated, as well as on the magnitude of problems with the imperfect sampling weights. For example, in the case of estimating y_{F,D_α} from imperfect weights, K is defined as $K = \text{cv}(\epsilon_i)\ \text{cv}(y_{i,D_\alpha})\ \text{cor}(\epsilon_i, y_{i,D_\alpha})$, where $\text{cv}(\cdot)$ is the coefficient of variation (the standard deviation divided by the mean), and $\text{cor}(\cdot,\cdot)$ is the correlation coefficient. K will tend to be large in magnitude when the imperfections in weights have a lot of variance ($\text{cv}(\epsilon_i)$ is large), when the quantity being estimated has large variance ($\text{cv}(y_{i,D_\alpha})$ is large), and when there is a strong relationship between the ϵ_i and the quantity being estimated ($\text{cor}(\epsilon_i, y_{i,D_\alpha})$). When the imperfect weights are exactly correct, $K = 0$.

The argument from Feehan and Salganik (2016a, Result D.10) can now be used to show that

$$\underbrace{\widehat{M}_\alpha}_{\substack{\text{network}\\ \text{survival}\\ \text{estimator}}} \rightsquigarrow \underbrace{M_\alpha}_{\substack{\text{true}\\ \text{death}\\ \text{rate}}} \times \underbrace{\frac{c_1}{c_2\ c_3}}_{\substack{\text{adapted}\\ \text{known}\\ \text{population}\\ \text{conditions}}} \times \underbrace{c_4}_{\substack{\text{frame}\\ \text{population}\\ \text{is complete}}} \times \underbrace{\frac{\tau_{F,\alpha}\delta_{F,\alpha}}{\eta_{F,\alpha}}}_{\substack{\text{reporting and}\\ \text{network}\\ \text{structure}}} \times \underbrace{\frac{(1+K_{F_1})}{(1+K_{F_2})}}_{\substack{\text{sampling}\\ \text{conditions}}}, \tag{C.8}$$

where $\rightsquigarrow$ means 'is consistent and essentially unbiased for', $K_{F_1} = \text{cv}(\epsilon_i)\text{cv}(y_{i,D_\alpha})\text{cor}(\epsilon_i, y_{i,D_\alpha})$ is the imperfect sampling index for y_{F,D_α}, and $K_{F_2} = \text{cv}(\epsilon_i)\text{cv}(y_{i,\mathcal{A}})\text{cor}(\epsilon_i, y_{i,\mathcal{A}})$ is the imperfect sampling index for $y_{F,\mathcal{A}}$.

Researchers who wish to assess how death rates estimated using network survival would be impacted by violations of any of the conditions required by the estimator can use Eq. C.8 to perform a sensitivity analysis by (i) assuming values or a range of values for c_1, c_2, c_3, c_4, $\tau_{F,\alpha}$, $\delta_{F,\alpha}$, $\eta_{F,\alpha}$, K_{F_1}, and K_{F_2}; and then (ii) using Eq. C.8 to determine the resulting values of M_α.

Worked example. For example, in order to create the lower-left panel of Figure 8, we set $\delta_{F,\alpha} = 0.5$ and $\eta_{F,\alpha}/\tau_{F,\alpha} = 1.5$ in Equation C.8. All of the other terms are set to $\frac{c_1}{c_2 c_3} = 1$, $c_4 = 1$, and $\frac{(1+K_{F_1})}{(1+K_{F_2})} = 1$. Rearranging Equation C.8, we find that in this situation, the expression

$$\widehat{M}_\alpha \frac{\eta_{F,\alpha}}{\tau_{F,\alpha}\delta_{F,\alpha}} \rightsquigarrow M_\alpha \tag{C.9}$$

will be consistent and essentially unbiased for the true death rate M_α. So we multiply the network survival estimates by $\frac{\eta_{F,\alpha}}{\tau_{F,\alpha}\delta_{F,\alpha}} = \frac{1.5}{0.5} = 3$.

D Tabular versions of results

This appendix provides tabular versions of Figure 4 (in Table D1), Figure 5 (in Table D2 and Table D3), Figure 6 (in Table D4), and Figure 7 (in Table D5).

Table D1: Estimated age-specific death rates using the acquaintance and meal tie definitions from the network survival study, and using the sibling history module of the DHS survey. Estimates are deaths rates per 1,000 person-years.

Tie definition	Sex	Age group	Estimate	95% CI
Acquaintance (2010-11; n=2,236)	Female	[15,25)	3.19	[2.12, 4.37]
Acquaintance (2010-11; n=2,236)	Female	[25,35)	2.97	[2.25, 3.82]
Acquaintance (2010-11; n=2,236)	Female	[35,45)	3.58	[2.43, 5.06]
Acquaintance (2010-11; n=2,236)	Female	[45,55)	5.82	[4.04, 8.07]
Acquaintance (2010-11; n=2,236)	Female	[55,65)	13.40	[9.30, 18.80]
Acquaintance (2010-11; n=2,236)	Male	[15,25)	3.96	[2.75, 5.59]
Acquaintance (2010-11; n=2,236)	Male	[25,35)	3.48	[2.58, 4.58]
Acquaintance (2010-11; n=2,236)	Male	[35,45)	7.97	[5.81, 10.54]
Acquaintance (2010-11; n=2,236)	Male	[45,55)	9.72	[7.17, 13.05]
Acquaintance (2010-11; n=2,236)	Male	[55,65)	20.69	[13.67, 31.73]
Meal (2010-11; n=2,433)	Female	[15,25)	5.71	[3.65, 7.93]
Meal (2010-11; n=2,433)	Female	[25,35)	4.08	[3.07, 5.28]
Meal (2010-11; n=2,433)	Female	[35,45)	6.15	[3.53, 9.48]
Meal (2010-11; n=2,433)	Female	[45,55)	8.03	[4.85, 12.42]
Meal (2010-11; n=2,433)	Female	[55,65)	10.04	[6.48, 14.82]
Meal (2010-11; n=2,433)	Male	[15,25)	4.30	[3.03, 5.80]
Meal (2010-11; n=2,433)	Male	[25,35)	4.57	[3.27, 6.12]
Meal (2010-11; n=2,433)	Male	[35,45)	7.48	[5.46, 9.79]
Meal (2010-11; n=2,433)	Male	[45,55)	9.05	[5.37, 14.22]
Meal (2010-11; n=2,433)	Male	[55,65)	15.40	[10.35, 22.93]
Sibling (2004-10; n=13,671)	Female	[15,25)	1.78	[1.41, 2.18]
Sibling (2004-10; n=13,671)	Female	[25,35)	3.61	[3.04, 4.18]
Sibling (2004-10; n=13,671)	Female	[35,45)	5.73	[4.89, 6.67]
Sibling (2004-10; n=13,671)	Female	[45,55)	4.63	[3.47, 5.88]
Sibling (2004-10; n=13,671)	Female	[55,65)	10.62	[6.03, 16.03]
Sibling (2004-10; n=13,671)	Male	[15,25)	2.18	[1.79, 2.58]
Sibling (2004-10; n=13,671)	Male	[25,35)	3.58	[2.99, 4.20]
Sibling (2004-10; n=13,671)	Male	[35,45)	6.41	[5.44, 7.45]
Sibling (2004-10; n=13,671)	Male	[45,55)	9.23	[7.22, 11.39]
Sibling (2004-10; n=13,671)	Male	[55,65)	19.60	[12.04, 28.19]

Table D2: Comparison between the estimated sampling distribution of the log age-specific death rate (log deaths per person-year) for the acqaintance network and for the sibling histories.

Sex	Age group	Mean difference in log(asdr estimate)	95% CI
Female	[15,25)	0.001	[0.000, 0.003]
Female	[25,35)	-0.001	[-0.002, 0.000]
Female	[35,45)	-0.002	[-0.004, 0.000]
Female	[45,55)	0.001	[-0.001, 0.004]
Female	[55,65)	0.003	[-0.004, 0.010]
Male	[15,25)	0.002	[0.001, 0.003]
Male	[25,35)	-0.0001	[-0.001, 0.001]
Male	[35,45)	0.002	[-0.001, 0.004]
Male	[45,55)	0.0005	[-0.003, 0.004]
Male	[55,65)	0.001	[-0.011, 0.014]

Table D3: Comparison between the estimated sampling distribution of the log age-specific death rate (log deaths per person-year) for the meal network and for the sibling histories.

Sex	Age group	Mean difference in log(asdr estimate)	95% CI
Female	[15,25)	0.004	[0.002, 0.006]
Female	[25,35)	0.0005	[-0.001, 0.002]
Female	[35,45)	0.0004	[-0.002, 0.004]
Female	[45,55)	0.003	[0.000, 0.008]
Female	[55,65)	-0.001	[-0.007, 0.006]
Male	[15,25)	0.002	[0.001, 0.004]
Male	[25,35)	0.001	[0.000, 0.003]
Male	[35,45)	0.001	[-0.001, 0.004]
Male	[45,55)	-0.0002	[-0.004, 0.005]
Male	[55,65)	-0.004	[-0.014, 0.006]

Table D4: Average number of deaths reported from each interview in Rwanda using the acquaintance and meal tie definitions from the network survival study and the sibling history module of the DHS.

Tie definition	Num. Reported deaths	Num. Interviews	Deaths / Interview
Acquaintance	1,681	2,259	0.74
Meal	932	2,404	0.39
Sibling (12 months)	124	13,671	0.01
Sibling (84 months)	1,197	13,671	0.09

Table D5: Estimated ${}_{45}q_{15}$ values, by tie definition and sex. The survey-based estimates have 95% confidence intervals, which come from the estimated sampling distribution of each estimator.

Tie definition	Sex	45q15	95% CI
Meal (2010-11)	Female	0.24	[0.19-0.30]
Sibling (2006-11)	Female	0.17	[0.15-0.20]
Acquaintance (2010-11)	Female	0.19	[0.15-0.23]
WHO (2012)	Female	0.21	
UNPD (2010-2015)	Female	0.19	
IHME (2011)	Female	0.21	
Meal (2010-11)	Male	0.26	[0.21-0.32]
Sibling (2006-11)	Male	0.28	[0.23-0.32]
Acquaintance (2010-11)	Male	0.26	[0.22-0.31]
WHO (2012)	Male	0.25	
UNPD (2010-2015)	Male	0.33	
IHME (2011)	Male	0.29	

E Network survival results for both sexes and tie definitions

Network survival estimates for adult death rates in Rwanda are shown in the main text (Figure 4). This appendix has additional plots that provide more detail about how the network survival death rates were estimated.

Our derivations in Section 3 and (in this online supplement) Section B show that network survival death rate estimates are built up from several components: the estimated number of connec-tions to deaths; the estimated personal network sizes; the estimated total number of deaths; and the estimated amount of exposure. The first part of this appendix has figures that show each of these components separately for all of the network survival death rate estimates from Rwanda: male death rates from the meal network (Figure E1); female death rates from the meal network (Figure E2); male death rates from the acquaintance network (Figure E3); and female death rates from the acquaintance network (Figure E4). The second part of this appendix has plots show-ing the age-specific death rates for both sexes and tie definitions that are not on a log scale (Figure E5).

Figure E1 shows detailed results for one case: estimated Rwandan male death rates from reports about the meal tie definition. Panel 1(a) shows, for each age group, the estimated total number of reports about deaths ($\widehat{y}_{F,D_\alpha}$, Eq. 6). Since each death can be reported multiple times, this quantity on its own is not enough to estimate the total number of deaths in the population. Panel 1(b) shows, for each age group, the estimated size of respondents' personal networks, which is used as an estimate for the visibility of deaths ($\widehat{\bar{\bar{d}}}_{F_\alpha,F}$, Eq. 7). Dividing the total estimated reports about deaths (Panel 1(a)) by the estimated visibility of deaths (Panel 1(b)) produces the estimated total number of deaths by age group ($\widehat{D}_\alpha$) shown in Panel 1(c). Panel 1(d) shows the estimated number of people in each age group ($\widehat{N}_{F_\alpha}$), which is used as an estimate of exposure; this quantity comes from the sampling design. The interpretation of Figures E2, E3, and E4 follow the same pattern as Figure E1.

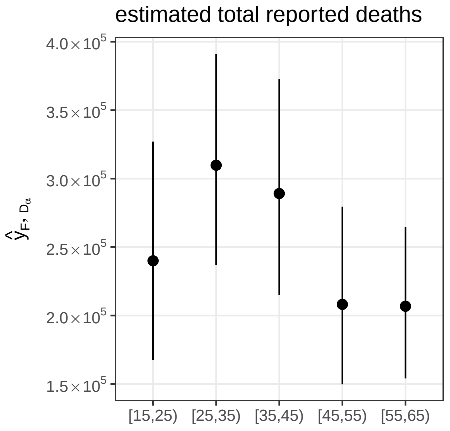

(a) $\widehat{y}_{F,D_\alpha}$

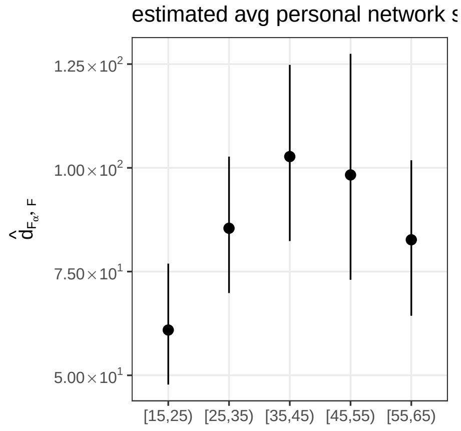

(b) $\widehat{\bar{d}}_{F_\alpha,F}$ (an estimate of $\bar{v}_{D_\alpha,F}$)

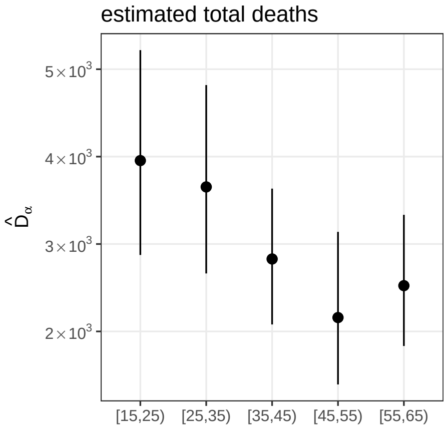

(c) $\widehat{D}_\alpha = \frac{\widehat{y}_{F,D_\alpha}}{\widehat{\bar{d}}_{F_\alpha,F}}$

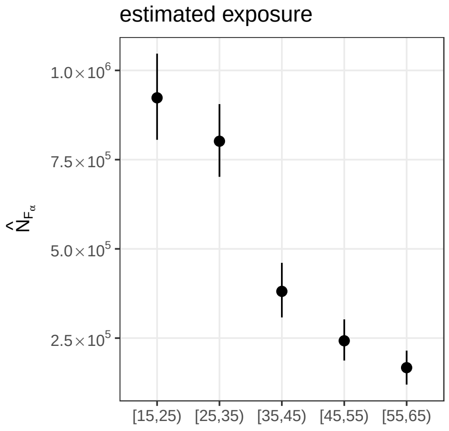

(d) $\widehat{N}_{F_\alpha}$ (an estimate of N_α)

Figure E1: Estimating components of age-specific death rates for Rwandan Males for 12 months prior to our 2011 survey using responses from the meal tie definition.

The average personal network size of survey respondents ($\widehat{\bar{d}}_{F_\alpha,F}$; Panel 1(b)), is used as an estimate of the visibility of deaths ($\bar{v}_{D_\alpha,F}$; i.e., the number of times each death could be reported). The estimated number of deaths in the population ($\widehat{D}_\alpha$; Panel 1(c)) is obtained by dividing estimated total reports about deaths ($\widehat{y}_{F,D_\alpha}$; Panel 1(a)) by the estimated visibility of deaths ($\widehat{\bar{v}}_{D_\alpha,F}$; Panel 1(b)). The estimated size of the frame population ($\widehat{N}_{F_\alpha}$) is used as an estimate of the population exposure N_α. Estimated age-specific death rates ($\widehat{M_\alpha}$; Figure 4) are obtained by dividing the estimated number of deaths ($\widehat{D}_\alpha$; Panel 1(c)) by the amount of exposure ($\widehat{N}_\alpha$; Panel 1(d)). Error bars show 95% confidence intervals; sampling uncertainty from each step is estimated using the rescaled bootstrap approach to account for the complex sample design (Rao et al., 1992; Rao and Wu, 1988).

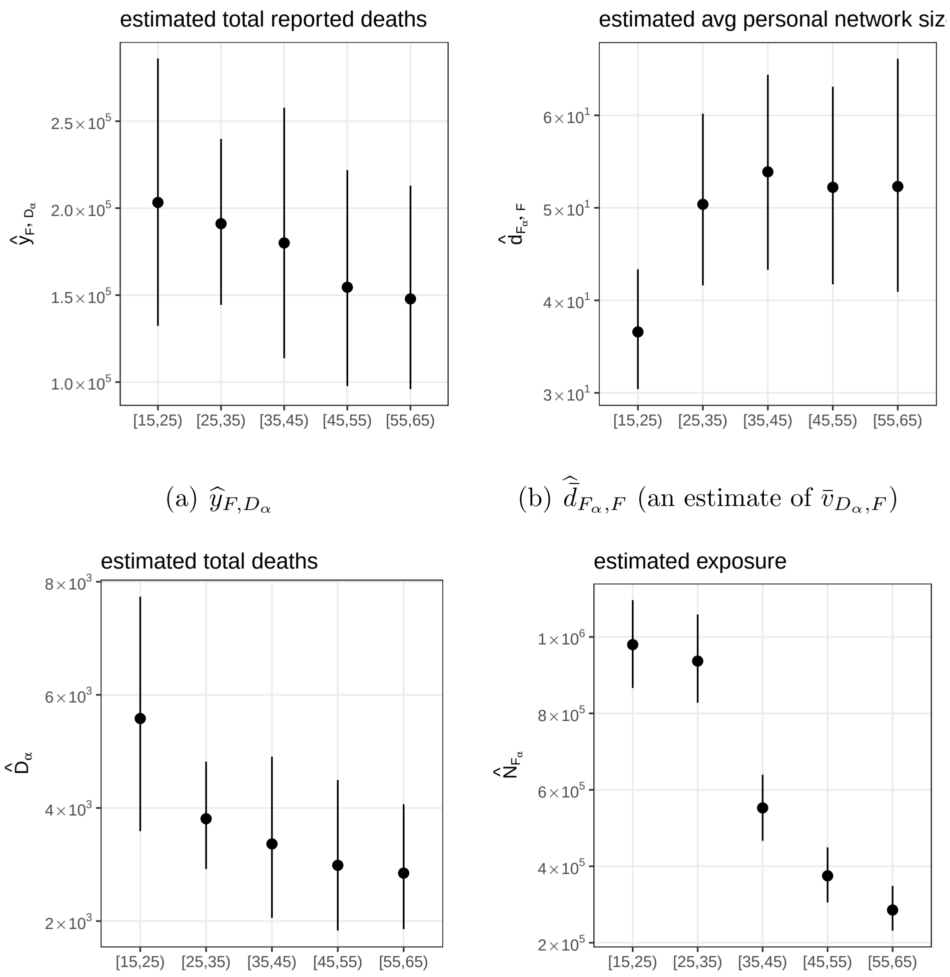

(a) $\widehat{y}_{F,D_\alpha}$

(b) $\widehat{\bar{d}}_{F_\alpha,F}$ (an estimate of $\bar{v}_{D_\alpha,F}$)

(c) $\widehat{D}_\alpha = \frac{\widehat{y}_{F,D_\alpha}}{\widehat{\bar{d}}_{F_\alpha,F}}$

(d) $\widehat{N}_{F_\alpha}$ (an estimate of N_α)

Figure E2: Estimating components of age-specific death rates for Rwandan females for 12 months prior to our survey using responses from the meal tie definition. The interpretation of this figure is analogous to Figure E1.

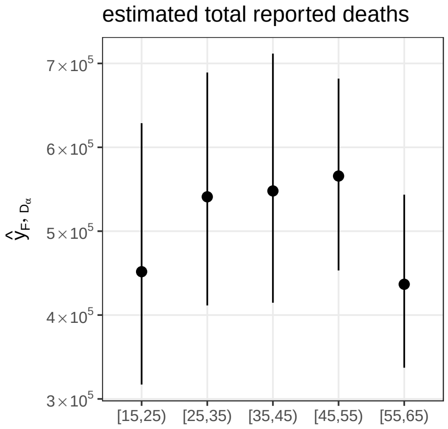

(a) $\widehat{y}_{F,D_\alpha}$

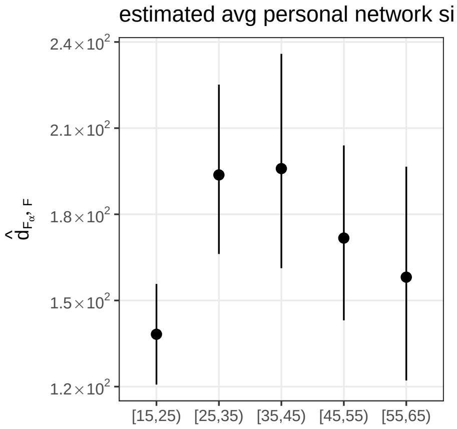

(b) $\widehat{\bar{d}}_{F_\alpha,F}$ (an estimate of $\bar{v}_{D_\alpha,F}$)

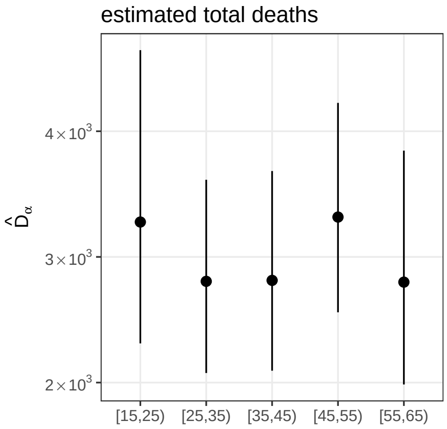

(c) $\widehat{D}_\alpha = \frac{\widehat{y}_{F,D_\alpha}}{\widehat{\bar{d}}_{F_\alpha,F}}$

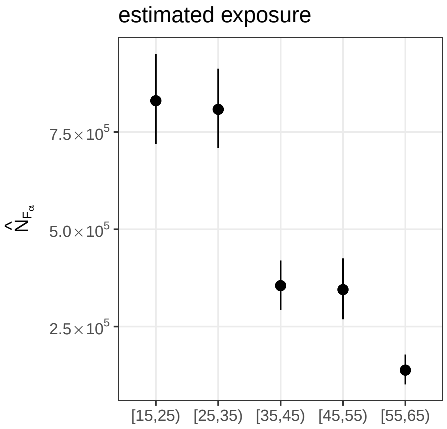

(d) $\widehat{N}_{F_\alpha}$ (an estimate of N_α)

Figure E3: Estimating components of age-specific death rates for Rwandan males for 12 months prior to our survey using responses from the acquaintance tie definition. The interpretation of this figure is analogous to Figure E1.

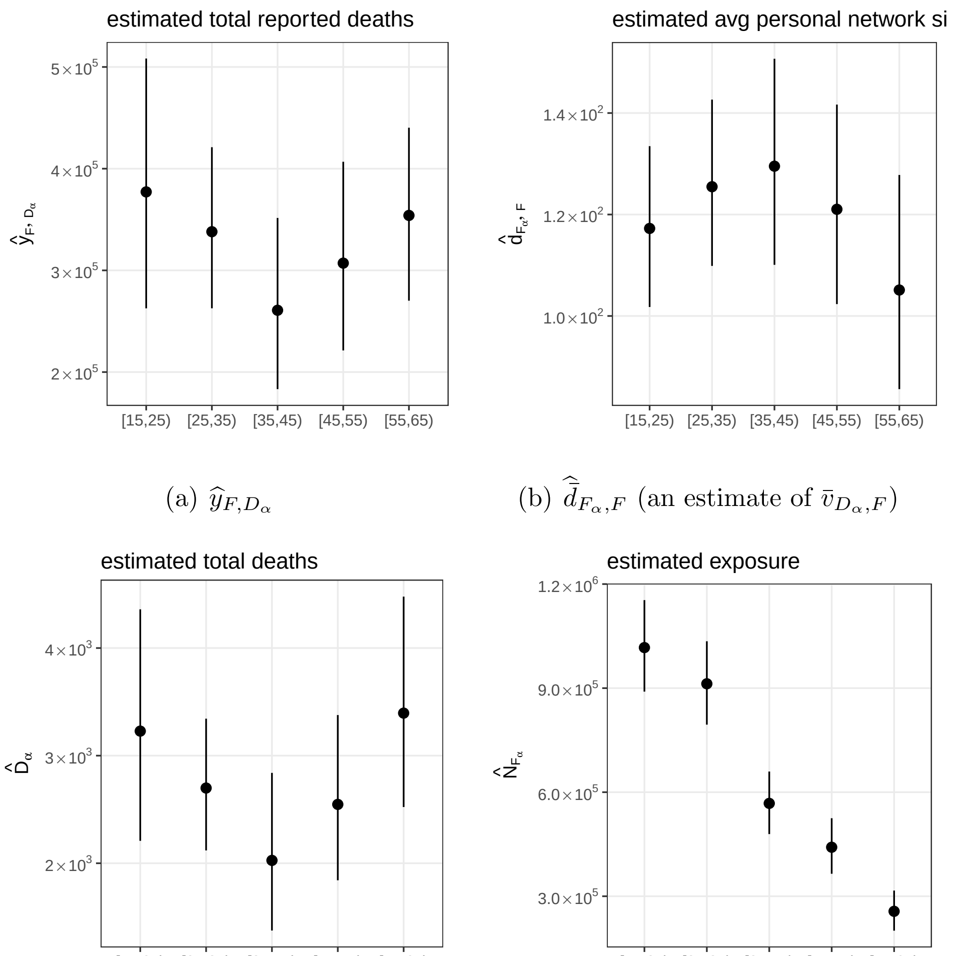

(a) $\widehat{y}_{F,D_\alpha}$

(b) $\widehat{\bar{d}}_{F_\alpha,F}$ (an estimate of $\bar{v}_{D_\alpha,F}$)

(c) $\widehat{D}_\alpha = \frac{\widehat{y}_{F,D_\alpha}}{\widehat{\bar{d}}_{F_\alpha,F}}$

(d) $\widehat{N}_{F_\alpha}$ (an estimate of N_α)

Figure E4: Estimating components of age-specific death rates for Rwandan females for 12 months prior to our survey using responses from the acquaintance tie definition. The interpretation of this figure is analogous to Figure E1.

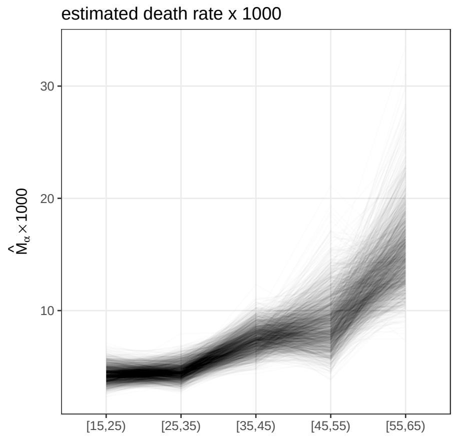

(a) Males, using the meal network.

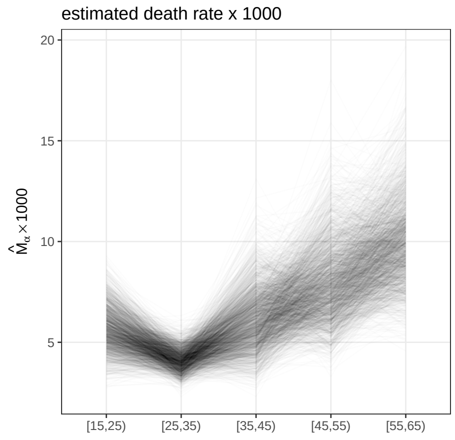

(b) Females, using the meal network.

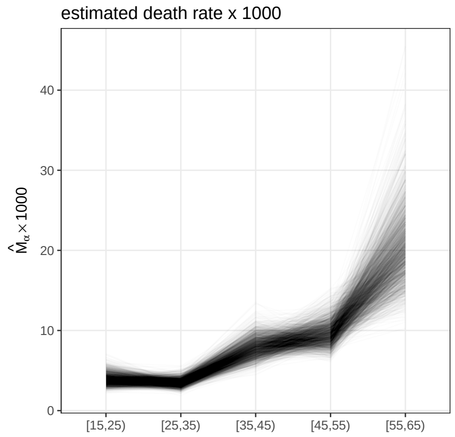

(c) Males, using the acquaintance network.

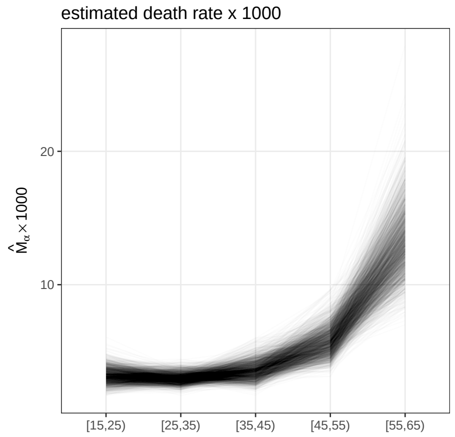

(d) Females, using the acquaintance network.

Figure E5: Estimated age-specific death rates for Rwandans for 12 months prior to our survey using responses from the meal tie definition (top row) and the acquain-tance tie definition (bottom row), for males (left column) and for females (right column). These plots are not on a log scale. Each line shows the result of one boot-strap resample; taken together, the lines show the estimated sampling uncertainty for each set of death rates.

F Comparison estimates

In this section, we provide more detail about the estimates we use to compare with network survival estimates. First, we describe how we constructed sibling survival estimates. Next, we give more information about the three organizations' estimates. We also show a comparison between network survival death rates and the death rates from the three organizations, providing a more granular comparison than the ${}_{45}q_{15}$ discussed in the main text.

F.1 Sibling survival estimates

In this section, we describe how we computed estimated adult death rates from the sibling histories in the 2010 Rwanda DHS using the direct sibling survival estimator. NISR et al. (2012) contains detailed information about the survey, and all of the data are freely available online through the DHS website[15].

Section 2 describes the considerable methodological debate over how to produce estimated death rates from DHS sibling histories. Our goal here was to construct the simplest direct sibling survival estimates possible. We therefore follow the recommendation of the offical *Guide to DHS Statistics* (Rutstein and Rojas, 2006) and the International Union for the Scientific Study of Population's *Tools for Demographic Estimation* (Moultrie et al., 2013) by using the original direct sibling survival estimator proposed by Rutenberg and Sullivan (1991). The estimator can be written

$$\widehat{M}_\alpha = \frac{\sum_{i \in s} \frac{1}{\pi_i} \sum_{k \in \sigma(i)} D_{k,\alpha}}{\sum_{i \in s} \frac{1}{\pi_i} \sum_{k \in \sigma(i)} N_{k,\alpha}}, \quad \text{(F.1)}$$

where $\widehat{M}_\alpha$ is the estimated death rate in demographic group α; s is the sample of survey respondents, π_i is respondent i's probability of inclusion from the sampling design; $\sigma(i)$ is the set of siblings that respondent i reports about; $D_{k,\alpha}$ is an indicator

[15] `http://dhsprogram.com/what-we-do/survey/survey-display-364.cfm`

variable for whether or not k died when in demographic group α, and $N_{k,\alpha}$ is the amount of time k spent alive in demographic group α.

We wanted to compare the network survival results (based on 12 months prior to the survey) to the sibling survival estimates. Therefore, our preference would be to compute sibling survival estimates for the 12 months prior to the survey. However, the left-hand panel of Figure F.1 shows that estimates for this time frame have too much sampling variation to be practically useful (and this is consistent with the sibling history literature; see Section 2). Since samples are not typically large enough to permit estimating yearly age-specific death rates using the estimator in Eq. F.1, in the results in the main text, we follow the recommendation of Rutstein and Rojas (2006) and Rutenberg and Sullivan (1991) by producing estimates for the 84 months (i.e., 7 years) prior to the survey.

F.2 Three organizations' estimates

Although estimates from organizations like the WHO, UNPD, and IHME are typically used to compare aggregate metrics of adult mortality like ${}_{45}q_{15}$ across countries, the organizations also produce age-specific death rate estimates. Figure F2 shows the estimated age-specific death rates from the two network survival estimates, the sibling survival estimates, and the age specific estimates for each organization.

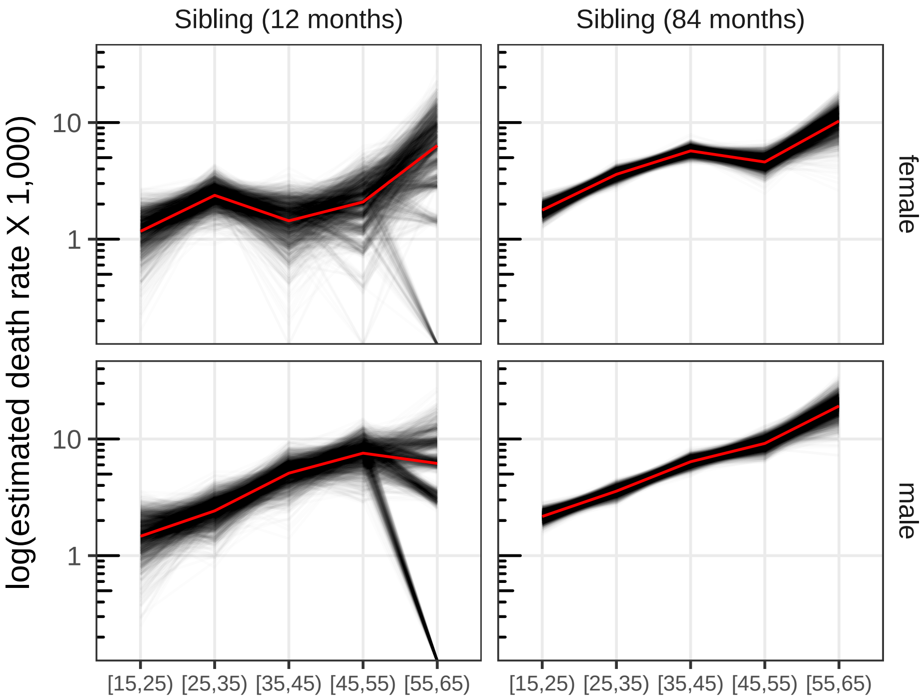

Figure F1: Comparison between sibling estimates based on deaths reported 12 months and 84 months before the interview. The estimates from 12 months before the interview are very imprecise, while the estimates from 84 months before the survey are much more stable. Therefore, we use the 84-month estimates when we compare to the network survival results in the main text.

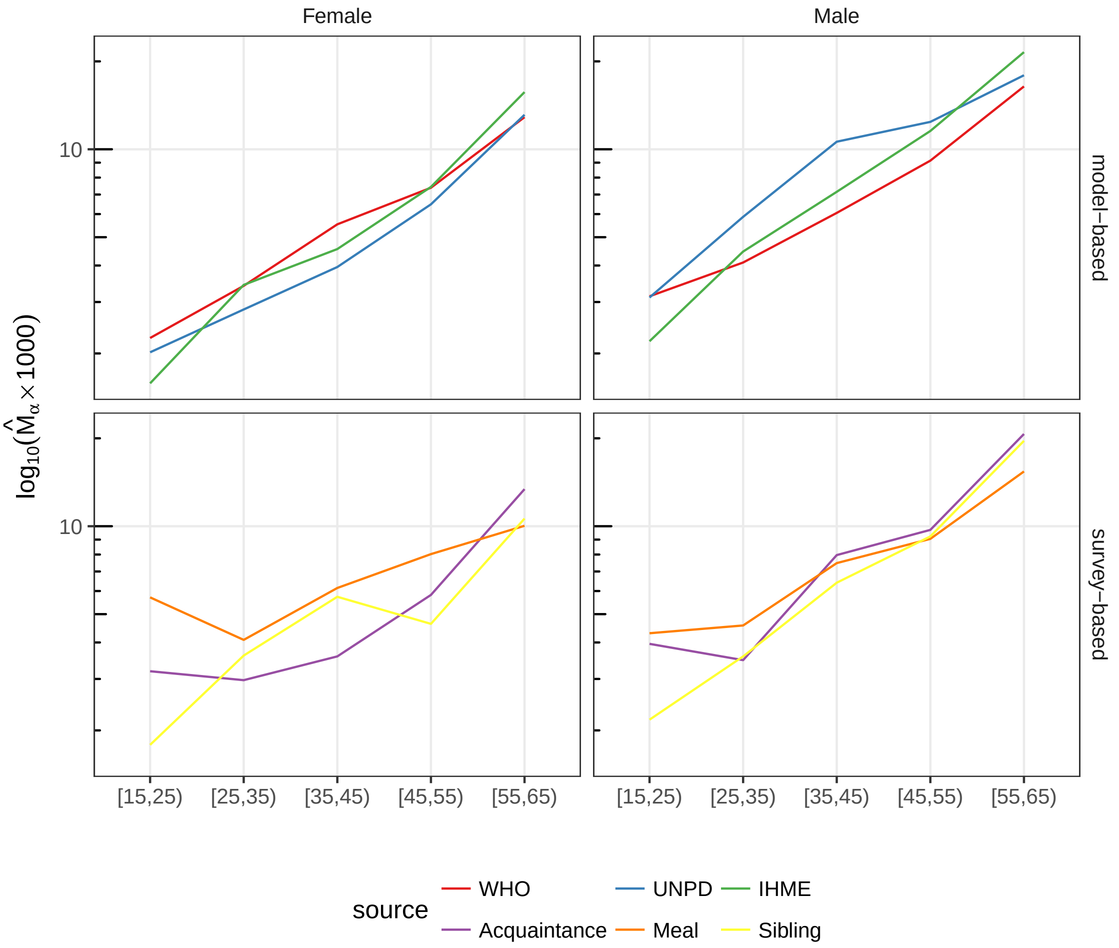

Figure F2: Comparison between network survival death rate estimates for two types of personal network, direct sibling survival death rates estimates from the 2010 Rwanda Demographic and Health Survey, and model-based estimates for age-specific death rates in Rwanda from three different organization. Sampling uncertainty for Acquaintance, Meal, and Sibling estimates are shown in Figure 4. Estimates from the WHO, UNPD, and IHME are model-based, so no comparable sampling-based uncertainty estimates are available.

G Issues related to the frame population

The frame population in our study (i.e., the set of people eligible to be interviewed) was all people age 15 and over. Some other surveys in developing countries, however, have different frame populations. For example, the frame populations in the Demographic and Health Surveys is typically women between 15 and 49 and men between 15 and 59. The difference between the frame population in our study and the frame populations typically used in the Demographic and Health Surveys naturally raises questions about the ability to embed the network reporting method as a module in other studies. Therefore, in this appendix we describe some of the analytic and practical issues raised by the choice of the frame population. We also artificially truncate our sample to match the Rwanda DHS respondents' age range (i.e., females 15-49 and males 15-59) and show that this truncation makes very little difference in our estimate of ${}_{35}q_{15}$. Further, in Section H of this appendix, we report descriptive plots showing how the data we collected varied by the age and sex of respondents.

The network reporting identity (Eq. 2) is true for any frame population. When that identity is re-arranged as in Eq. 4, it reveals the key qualitative insight of our approach: estimating the number of deaths from the number of reports of deaths requires correctly adjusting for the visibility of deaths. Thus, the key issue with the network reporting method is estimating the visibility of deaths to the frame population. In this study, we used the average personal network size of respondents in demographic group α as an estimate of the average visibility of deaths in demographic group α to the frame population. This exact approach is not possible if the frame population is more restricted; for example, if the frame population was restricted to women between 15 and 49, we would not have information to estimate the average personal network size of men between 15 and 29.

We see two different general approaches for the problem of estimating the visibility of deaths when the frame population is not all people age 15 and over. First, researchers can make additional assumptions. Researchers could, for example, make assumptions about the relationship between the personal network size of men and women or

between young people and old people. (Naturally, researchers adopting this approach would need to assess the sensitivity of their estimates to these assumptions.) Second, researchers can collect additional data to directly estimate the visibility of deaths to the frame population. In other words, if the frame population is women between 15 and 49, then researchers could collect information to estimate the visibility of deaths to women between 15 and 49. We see this second approach as more promising and some ideas in this direction might be taken from the generalized network scale-up method, which also involves two data collections (Feehan and Salganik, 2016a).

Additionally, as a rough empirical check of how our results in this study might have been impacted if we had a different frame population, we artificially truncate our sample to women between age 15 and 49 and men between ages 15 and 59 to match the frame population for the 2010 Rwanda DHS. This procedure First, Figure G5 shows that the full sample and truncated sample reported similar number of deaths per interview. Second, Figure G2 shows that the full sample and the truncated sample produce similar estimates of ${}_{35}q_{15}$. Note that we estimated ${}_{35}q_{15}$ instead of ${}_{45}q_{15}$ because estimating ${}_{45}q_{15}$ requires information about the visibility of deaths of people aged 50 to 65 and our study was not designed to estimate this quantity using only the subset of respondents under age 50.

Finally, as suggested by a reviewer, we investigate the relationship between the age of the reported deaths and the age of the respondents who reported them. Figure G3 shows the age distribution of reported deaths by the age range of respondents; further, Table G1 shows the number of reported deaths by tie definition, respondent age range, and death age range. Network survival respondents who are the same age as DHS respondents report deaths among people over 50 about one third of the time (meal: 0.33, acquaintance: 0.38); network survival respondents who are older than DHS respondents report deaths among people over 50 just under two-thirds of the time (meal: 0.57, acquaintance: 0.62). Figure G4 shows the relationship between the age of the survey respondent and the age of the reported death, for all of the deaths reported using both tie definitions in our survey, and using the DHS sibling histories. Three main conclusions emerge from Figure G3, Table G1, and

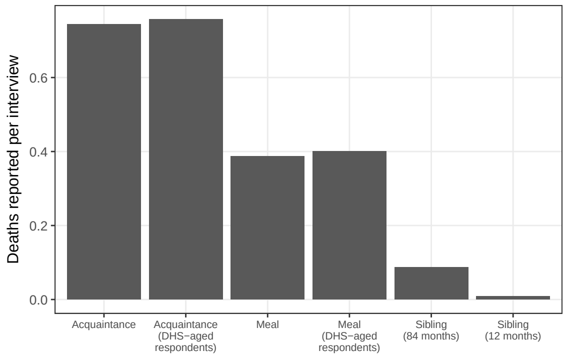

Figure G1: Average number of deaths reported from each interview in Rwanda using the acquaintance and meal tie definitions from the network survival study, and using the sibling history module of the DHS survey. Results from the network survival study are shown for all respondents, and for DHS-aged respondents (women 15-49 and men 15-59).

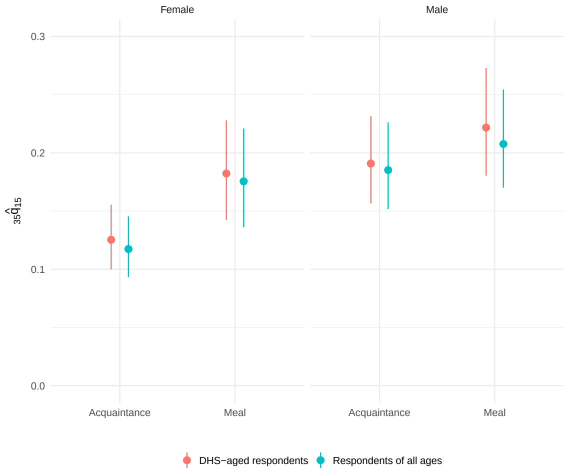

Figure G2: Comparison between network survival estimates of ${}_{35}q_{15}$ for males and for females using all respondents and using only DHS-aged respondents (women 15-49 and men 15-59).

Figure G4: (1) deaths over age 50 are reported both by network survival respondents who are in age ranges typically interviewed by the DHS, and also by network survival respondents who are older than typical DHS interviewees; (2), network survival respondents who are older than typical DHS interviewees report a greater fraction of deaths over age 50 than network survival respondents in typical DHS age ranges; and (3), using the meal and acquaintance tie definitions, network survival respondents of a given age appear to report deaths across a wider range of ages than sibling survival respondents.

Table G1: Number of deaths reported in Rwanda using the acquaintance and meal tie definitions from the network survival study, by age range of respondent and age of reported death.

Tie definition	Respondent age	Reported death age	Num. reported deaths
Acquaintance	older than DHS	death <50	123
Acquaintance	older than DHS	death 50+	197
Acquaintance	same as DHS	death <50	1,375
Acquaintance	same as DHS	death 50+	854
Meal	older than DHS	death <50	71
Meal	older than DHS	death 50+	95
Meal	same as DHS	death <50	753
Meal	same as DHS	death 50+	373

In conclusion, the network reporting method can be used for any frame population, but researchers using a frame population other than all adults would need to make some slight modifications from the approach taken in this paper. We think that this represents an important area for future research.

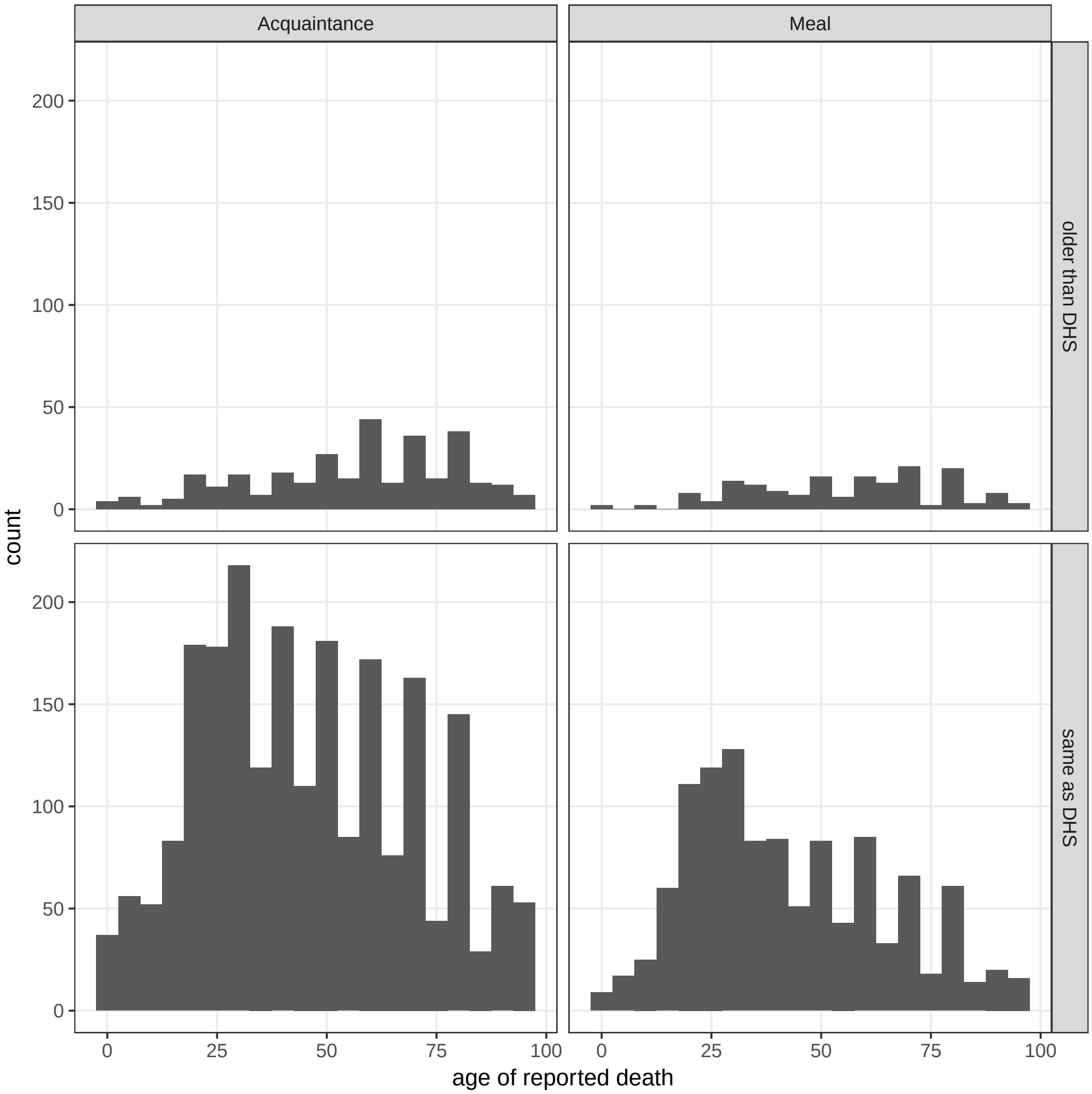

Figure G3: Distribution of the ages of reported deaths by tie definition and by whether or not respondents are in the age ranges typical of DHS surveys (females 15-49 and males 15-59). Bins have width 5 years; this figure does not use the sampling weights.

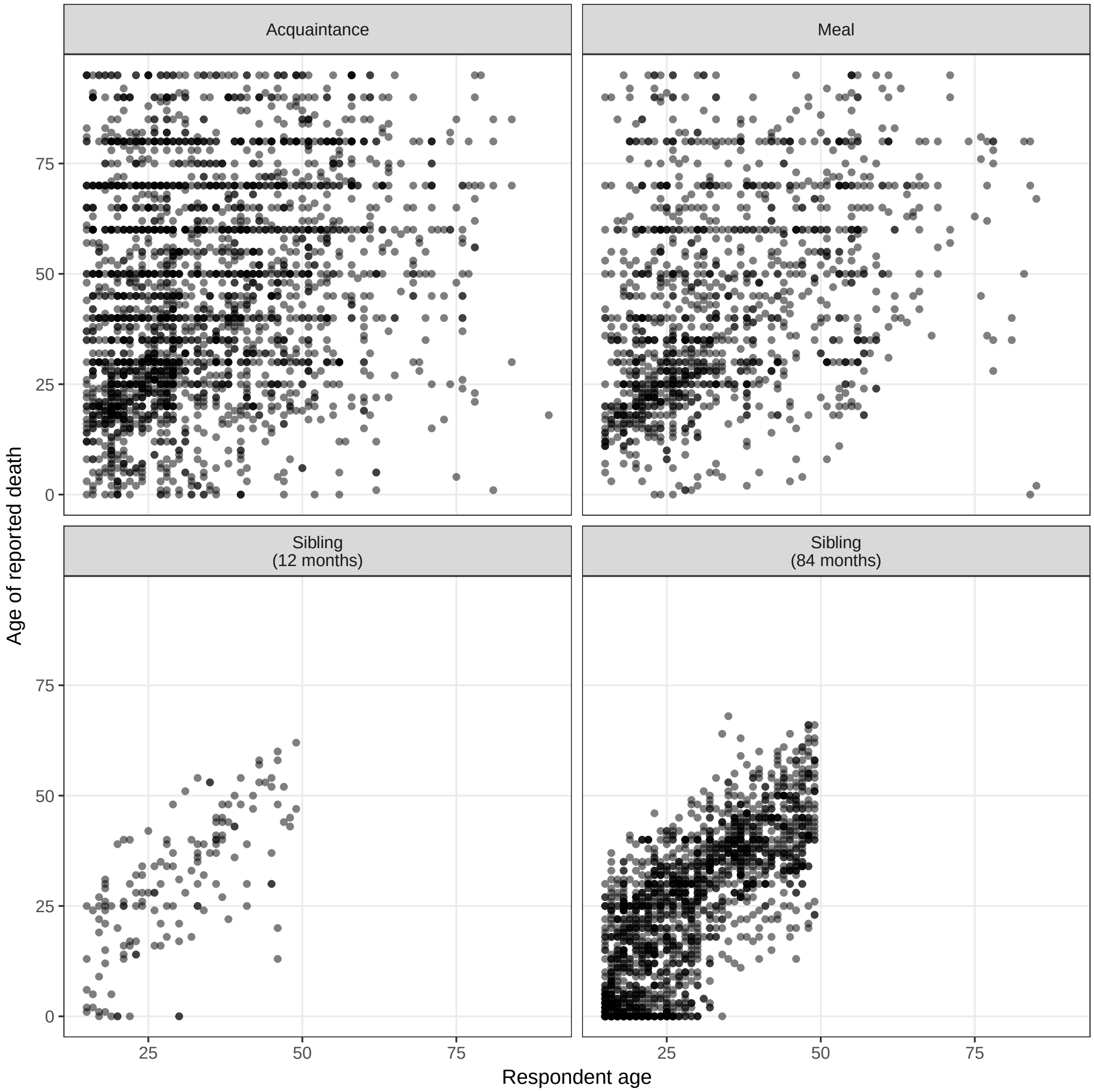

Figure G4: Age of reported death versus age of survey respondent for the acquaintance and meal tie definitions in our network survey, and from the sibling history of the DHS. There is one point for each reported death, so survey respondents who report more than one death contribute more than one point to the plot. The Rwanda DHS only asked the sibling histories of women, so respondents for the sibling method are all under 50.

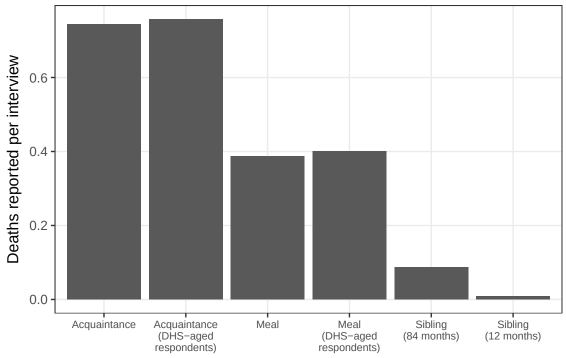

Figure G.5: Average number of deaths reported from each interview in Rwanda using the acquaintance and meal tie definitions from the network survival study, and using the sibling history module of the DHS survey. Results from the network survival study are shown for all respondents, and for DHS-aged respondents (women 15-49 and men 15-59).

H Descriptive plots

This appendix provides additional descriptive plots related to the network reporting method and the sibling survival method. In particular, we include plots related to reports about deaths in both methods (Sec. H.1) and reports of connections to groups of known size in the network reporting method (Sec. H.2).

H.1 Reports about deaths

Figure H1 shows the distribution of the number of deaths reported by each survey respondent. Two main findings emerge from this plot: 1) as reported in the main paper, the network reporting method (both tie definitions) collects more deaths per interview than the sibling method, even when the sibling reports are taken over a 7 year time period; 2) in all cases, the distributions seem to vary smoothly suggesting that the higher number of reports in the network survival method are not driven by a small number of extreme outliers.

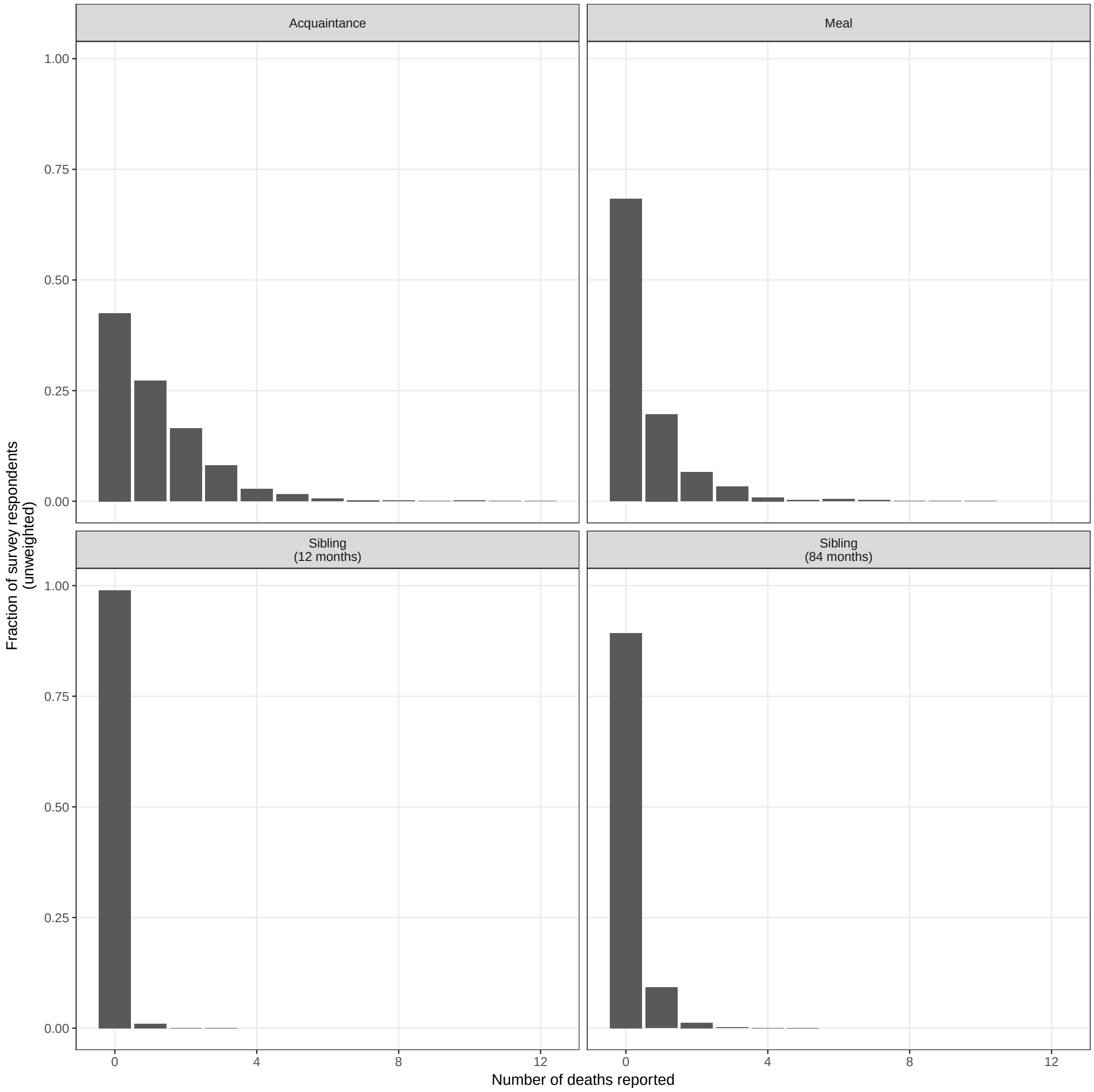

Figure H1: Distribution of the number of deaths reported by survey respondents to both types of personal network, and to the sibling histories using two time windows (12 months and 84 months). Each panel shows the unweighted fraction of respondents who reported each possible number of deaths.

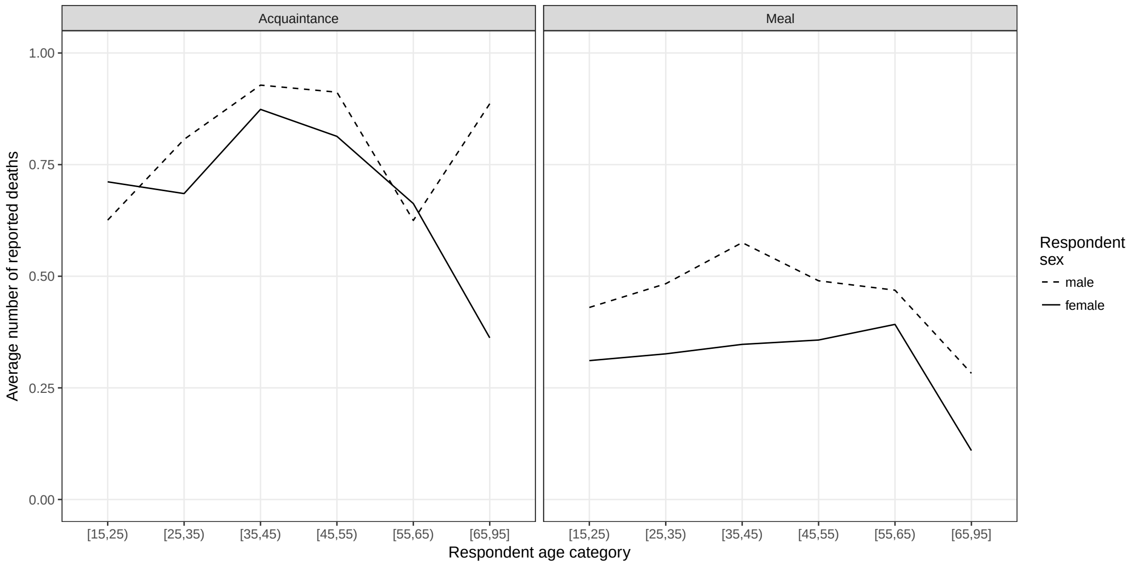

Figure H2: Average number of adult deaths reported for each tie definition, by age and sex of survey respondents.

Further, as described in Section G, future studies might use a frame popu-lation more restricted than our frame population of all adults. Therefore, Figure H2 shows the average number of adult deaths reported by the age and sex of survey respondents. Two observations emerge from this figure: first, for the acquaintance network, there appears to be a U-shaped relationship between respondent age and the average number of deaths reported. Second, for both tie definitions, males ap-pear to report more deaths, on average, then females. Figure H3 shows the average number of adult deaths reported by age of women who responded to the DHS sibling history module. The main observation to emerge from this figure is that the number of sibling deaths reported appears to increase with respondent age. Taken together, one possible explanation for the difference between the reporting patterns in sibling networks (Figure H3) and the reporting patterns in meal and acquaintance networks (Figure H2) is that siblings tend to be more similar to respondents in terms of age than acquaintances or meal partners.

Additionally, Figure H4 shows the distribution of the ages of reported deaths from

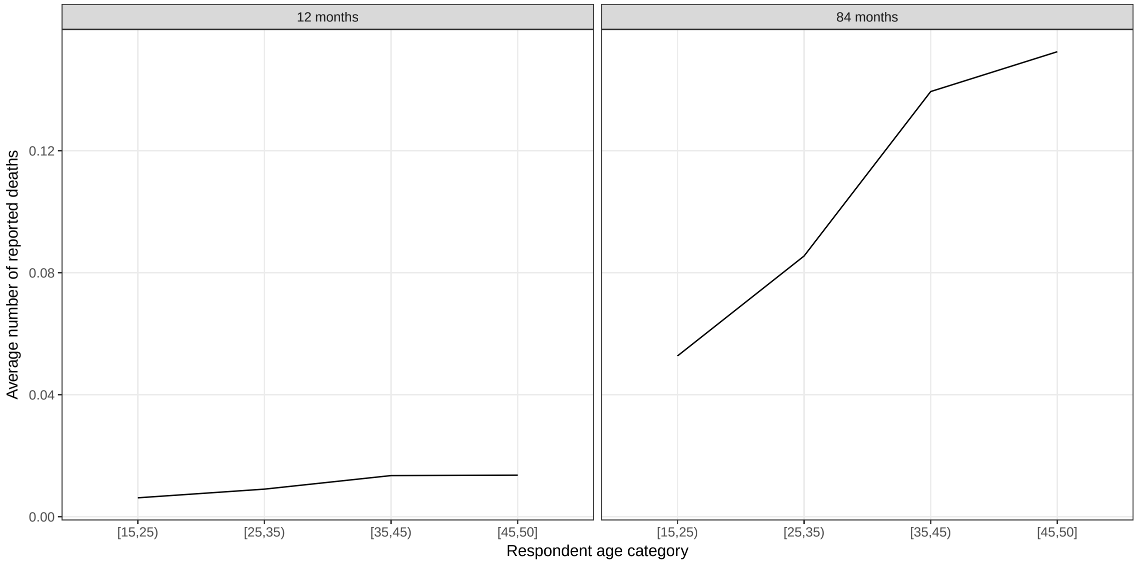

Figure H3: Average number of adult deaths reported for 12 months before the interview (left panel) and for 84 months before the interview (right panel), by age of women responding to the DHS sibling histories. Note that the last age group ends at 50, since the DHS only asked the sibling history module of women up to age 50.

the two personal networks and from the sibling reports for two different time periods as a function of respondent level of education. Several observations emerge from this plot: first, reports appear to be more heaped for less educated respondents; second, there appears to be considerably more heaping for the network reports, when compared to the sibling reports over an 84 month time period. The small number of deaths for the sibling reports over a 12 month time period make it very hard to draw any conclusions.

Finally, in order to explore whether the sibling survival method and the network survival method could be impacted by interviewer effects, we plot the number of reported deaths by interviewer. Figure H5 shows the average number of reported deaths per interview by interviewer and by tie definition from our study. And, similarly, Figure H6 shows the average number of reported deaths per interview by interviewer and by time window for deaths from the 2010 Rwanda DHS sibling histories. These figures do not show strong evidence of interviewer effects, but neither our survey nor the DHS were specifically designed to measure possible interviewer effects. We hope that this topic will be studied in future research.

H.2 Connections to groups of known size

The network survival method (as we operationalized it in this study) asked respondents about their connections to groups of known size in order to estimate their personal network size. Figure H7 shows the distribution of the number of reported connections to each group of known size; and Figure H8 and Table H1 show the relationship between the average number of reported connections to each known population and the size of each known population. As expected, respondents report more connections to larger groups, a common pattern in studies using the network scale-up method. The correlation between the average number of reported connections and the total size of the known populations is 0.66 for the Acquaintance tie definition and 0.86 for Meal tie definition. For the Acquaintance network results, Figure H8 shows that one group (teachers, 3.5 average reported connections) appears to fall well above

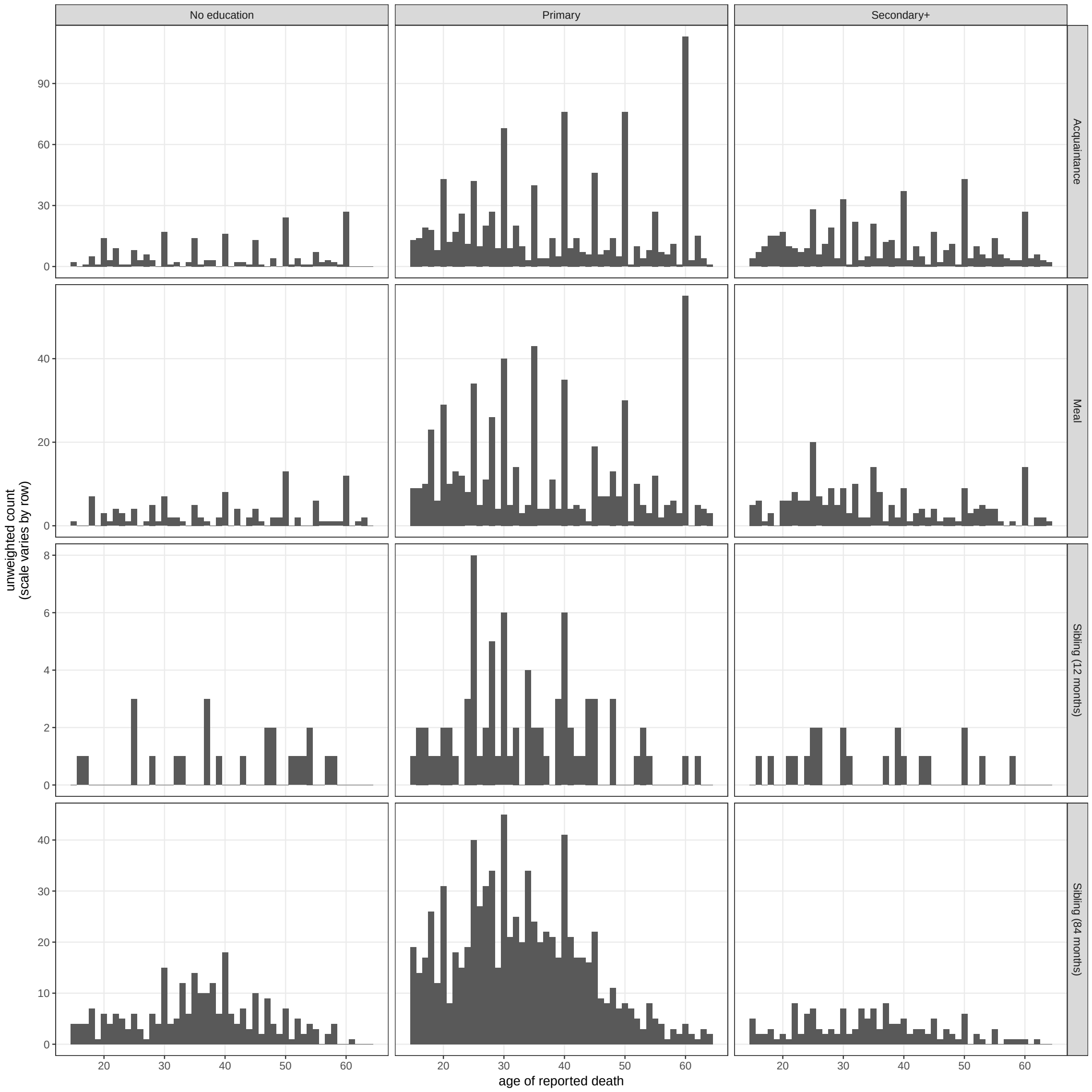

Figure H4: Distribution of the ages of reported deaths by single year of age from the two personal networks, from sibling reports 12 months prior to the survey, and from sibling reports 84 months prior to the survey (rows), and by education of survey respondent (columns). Note that the scale varies by row, since the total number of deaths reported varies considerably between the different tie definitions.

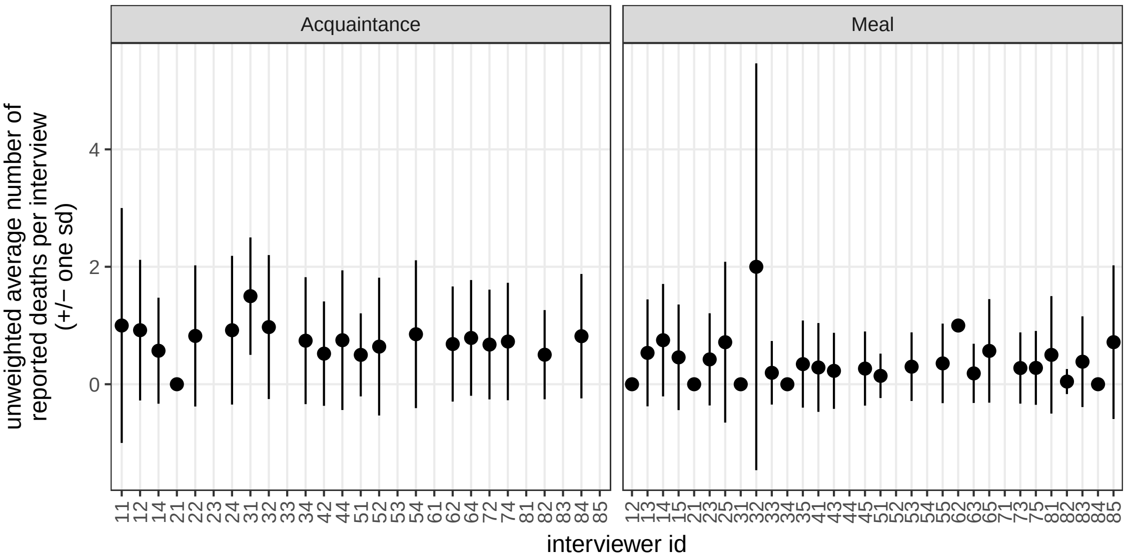

Figure H5: Average (+/- one s.d.) in the number of reported deaths per interview, by interviewer and by tie definition for the two personal networks. Note that in-terviewer id 32 only conducted 3 interviews using the meal definition, which may explain the large standard deviation around that observation.

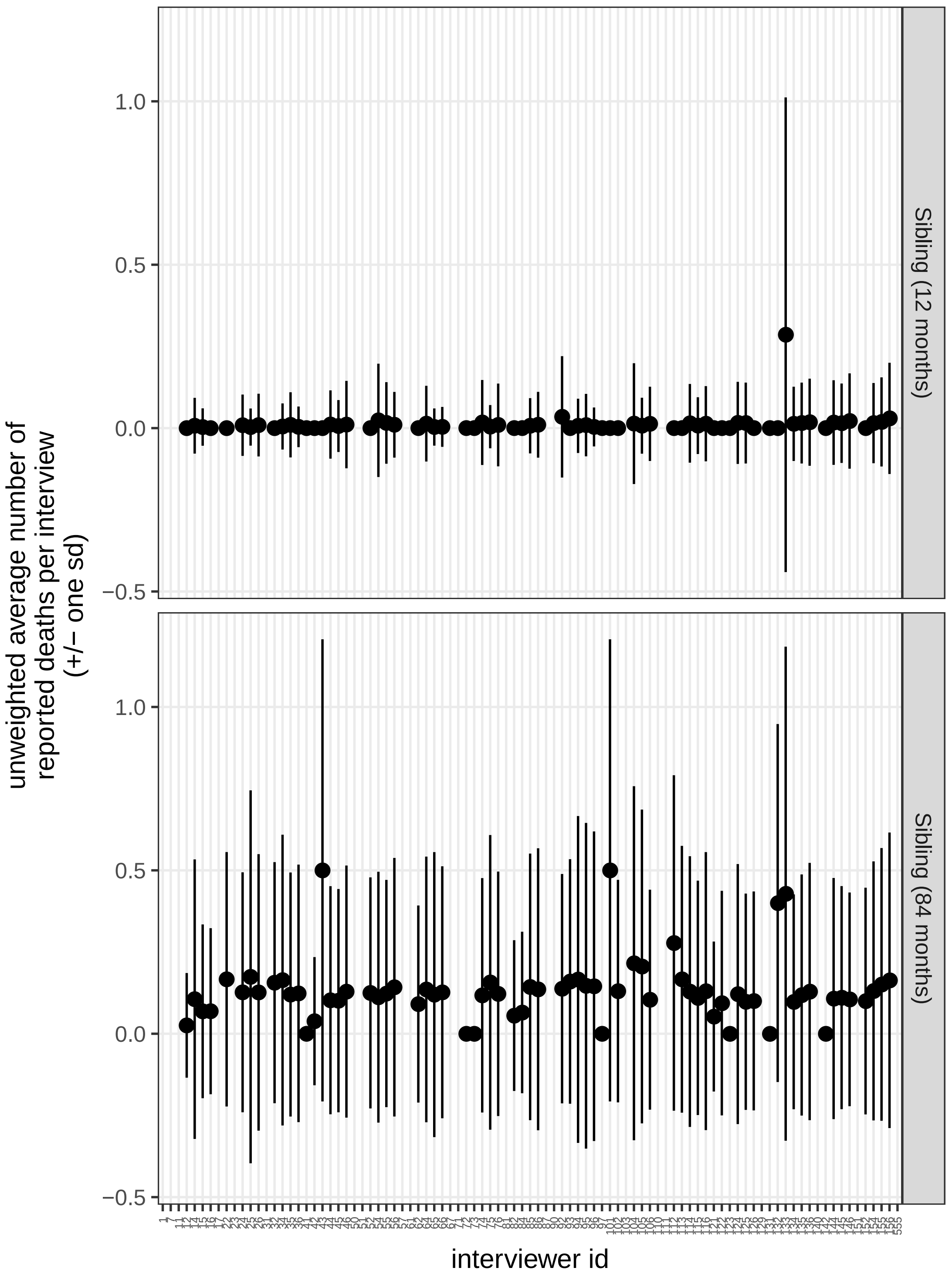

Figure H6: Average (+/- one s.d.) in the number of reported deaths per interview, by interviewer and by length of reporting interval for deaths from the Rwanda DHS sibling histories. Note that some interviewers conducted very few interviews, which may explain wide standard deviations in reports for interviewer id 43 (2 interviews), id 101 (2 interviews), and id 132 (5 interviews).

the pattern set by the remaining known populations. We cannot say what causes this deviation, but one possibility is that teachers have larger acquaintance networks than the average Rwandan.

Table H1: Average number of reported connections and known group size for each of the known populations.

Group	Total size	Avg. Connections (Acquaintance)	Avg. Connections (Meal)
Priest	1,004	0.35	0.11
Nurse or doctor	7,807	1.32	0.42
Twahirwa	10,420	0.68	0.26
Mukandekezi	10,520	0.56	0.18
Nyiraneza	21,705	0.85	0.30
Male community health worker	22,000	1.47	0.74
Ndayambaje	22,724	0.93	0.36
Murekatete	30,531	0.94	0.36
Nsengimana	32,528	0.95	0.40
Mukandayisenga	35,055	0.67	0.29
Widower	36,147	0.91	0.61
Ndagijimana	37,375	0.90	0.36
Bizimana	38,497	1.14	0.46
Nyirahabimana	42,727	0.84	0.30
Teacher	47,745	3.50	1.14
Nsabimana	48,560	1.23	0.50
Divorced man	50,698	0.50	0.31
Mukamana	51,449	1.29	0.45
Incarcerated	68,000	1.53	0.38
Woman who smokes	119,438	2.20	1.02
Muslim	195,449	2.21	1.04
Woman who gave birth last 12 mo.	256,164	2.87	1.99

Figure H9 shows the results of internal consistency checks that provide further evidence about the plausibility of the reported connections to groups of known size. These internal consistency checks are based on taking each known population, pretending its size is not known, estimating network size using the remaining known populations, and then using those estimated network sizes to predict the size of the held-out known population (see Feehan et al. (2016) for more details). Almost all of the hold-out estimates shown in Figure H9 lie close to the diagonal line, suggesting that reported connections to the groups of known size are internally consistent; how-

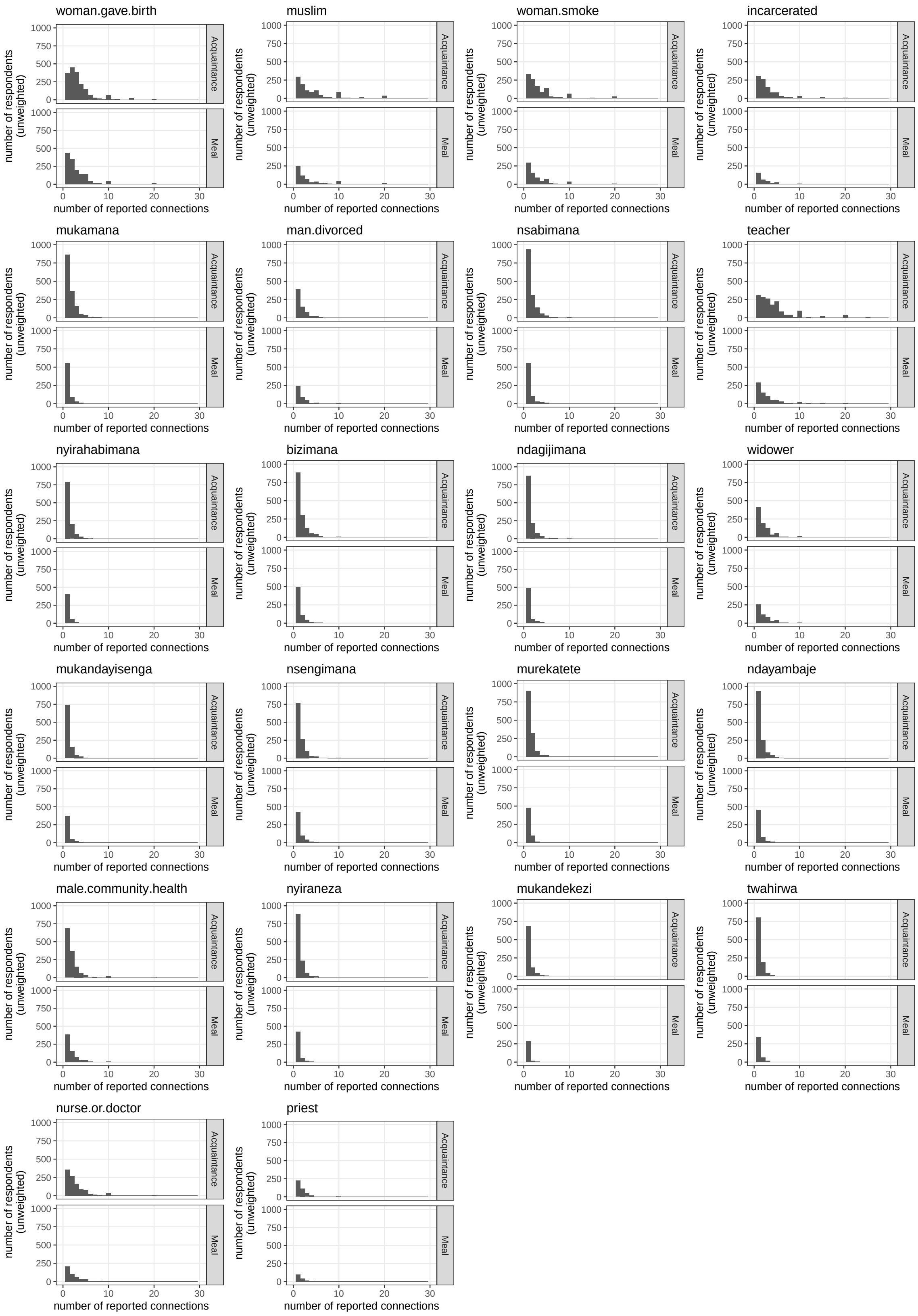

Figure H7: Distribution of the number of reported connections to each group of known size for the meal and acquaintance networks. Panels are sorted so that the largest known population is at the top-left and the smallest is on the bottom-right.

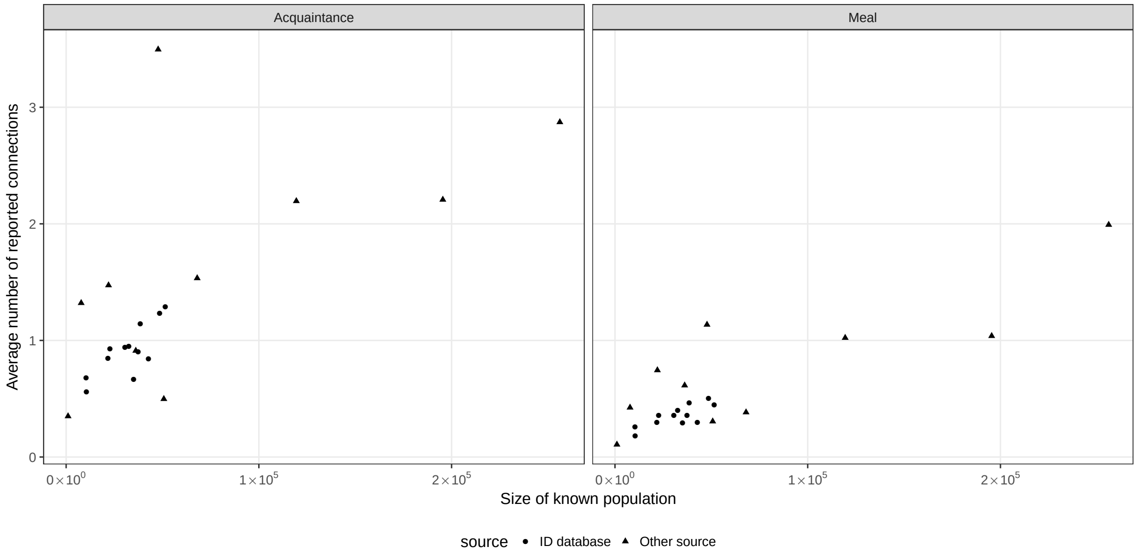

Figure H8: Average number of connections reported by survey respondents using the acquaintance network (left panel) and the meal network (right panel) versus the size of each known population. For both tie definitions, there is a strong positive relationship between the average reported connections and the size of known populations.

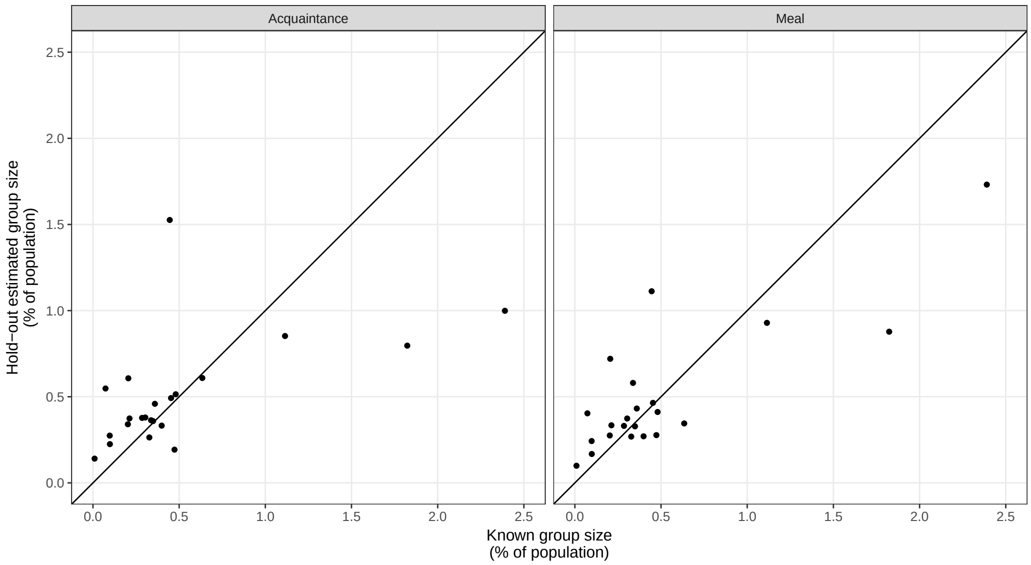

Figure H9: Results of internal consistency checks for the acquaintance and meal tie definitions in Rwanda. Each point in the plot represents a single known population. Taking divorced men as an example, the hold-out estimate is calculated by (1) estimating personal network size using all known populations *except* divorced men; (2) using number of reported connections to divorced men together with the hold-out estimates of personal network size to estimate the number of divorced men; and (3) comparing the hold-out estimate for the number of divorced men to the known size of that group. This exercise is repeated once for each group of known size, and for each tie definition. If these hold-out estimates were perfectly accurate, then all of the points in the two panels would lie along the diagonal lines.

ever, two groups (women who gave birth in the past 12 months and Muslims) both of appear to be underestimated in the hold-out checks.

Finally, Figure H10 plots, for each age group, sex, and tie definition, how the estimated average personal network size would change if each known population was not used. Figure H10 shows that estimated average personal network size appears not to be dramatically affected by the decision to include any particular group of known size. To be clear, we consider Figure H10 to be a heuristically useful diagnostic plot. However, it is important to note that a desirable set of known populations is one that satisfies the conditions required by the adapted known population estimator

(Result A.1). Such a set of known populations could include individual groups whose removal appreciably impacts estimated average personal network size.

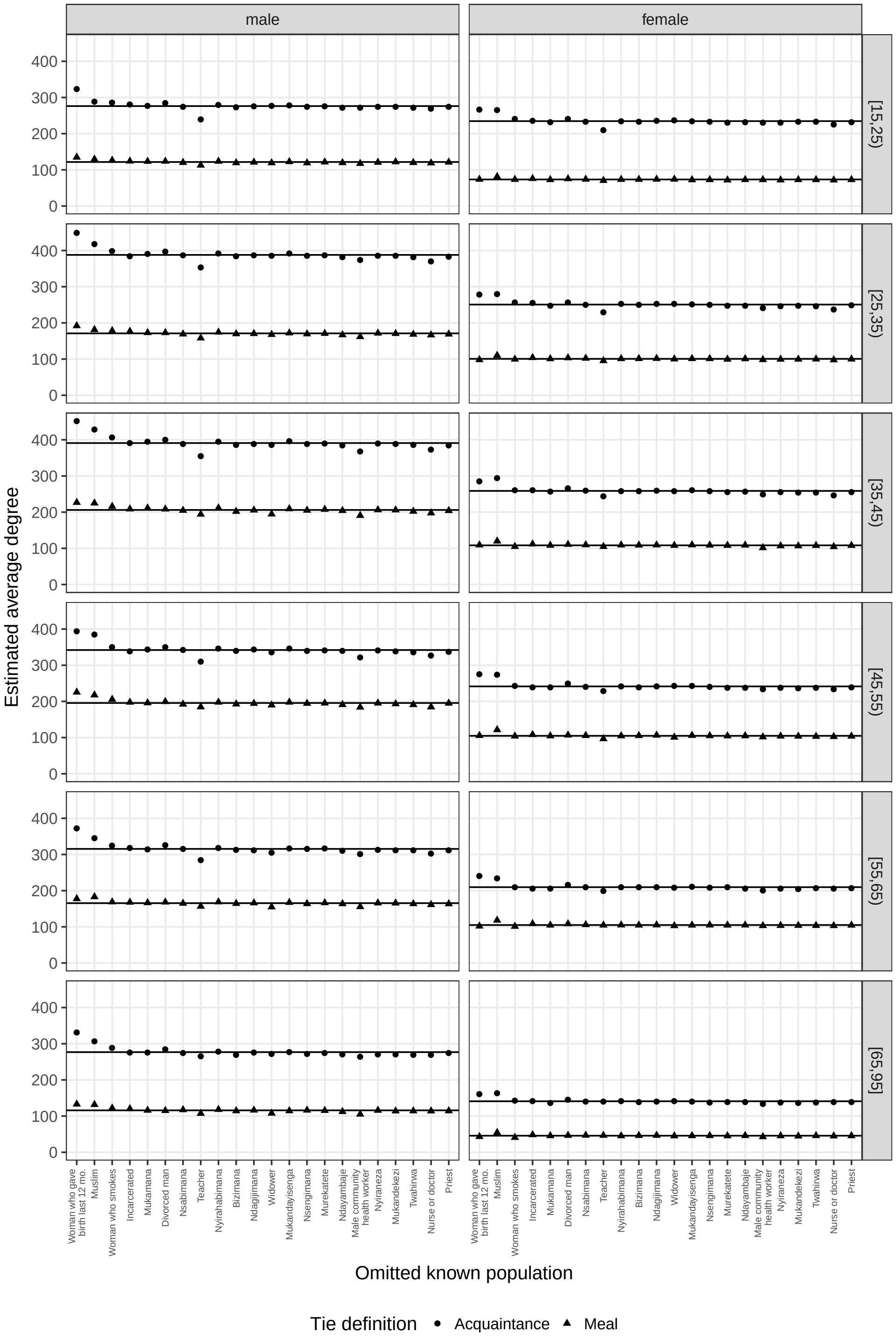

Figure H10: Impact of each known population on estimating average personal network size, by sex, age group, and tie definition. The horizontal line shows the estimated average personal network size using all of the known populations, and each point shows the estimated personal network size calculated using all of the known populations except for the one listed on the x axis. The distance between each point and the horizontal line shows how different the estimated personal network size would be if the corresponding known population was not used. The groups are shown on the x axis in order of their total size from largest to smallest.

I Network survival survey instrument

In this appendix, we reproduce an excerpt of the English translation of the survey instrument that we used for the meal tie definition, and we comment on its design. All of the survey materials—including the original Kinyarwanda instruments for both the meal and tie definition, as well as their English translations—are freely available from the DHS website[16].

We had to pay careful attention to constructing the wording of the question that asked respondents to report about deaths (Q226). Both tie definitions in our study were based on interactions (Table 1)—either contact (for the acquaintance definition) or sharing a meal or drink (for the meal definition). Of course, people who have died cannot continue to interact with others. Therefore, in this section, we generalize the framework introduced in the main text to account for tie definitions where people's degree could change daily (e.g., tie definitions that are based on interactions). Without loss of generality, we will consider the meal definition.

When asking respondents about connections to people in the groups of known size, we ask about people who the respondent has shared a meal with in the 12 months before the interview. When asking about people who have died, we asked about people where: (i) the person died in the 12 months before the interview; and (ii) the person shared a meal with the respondent *in the 12 months before death* (see Q226). In this situation, the decedent network condition needs to be generalized into the *dynamic decedent network condition.*

The decedent network condition discussed in main text and in Result B.2 says that:

$$\bar{d}_{D_\alpha,F} = \bar{d}_{F_\alpha,F}, \tag{I.1}$$

where $\bar{d}_{D_\alpha,F}$ is the average degree of people who have died in group α and $\bar{d}_{F_\alpha,F}$ is the average degree of frame population members in group α.

[16] http://dhsprogram.com/what-we-do/survey/survey-display-422.cfm

The analogous dynamic decedent network condition says that:

$$\frac{1}{D_\alpha} \sum_{i \in D_\alpha} \Delta_{i,F}^{\delta(i)} = \frac{1}{N_{F_\alpha}} \sum_{i \in F_\alpha} \Delta_{i,F}^{\omega}, \tag{I.2}$$

where $\Delta_{i,F}^{t}$ is the number of personal network connections from i to the frame population F at time t; $\delta(i)$ is the day in which i died (for $i \in D_\alpha$); and ω be the date of the survey (we will assume all of the interviews take place on the same date). For example, the dynamic decedent network connection says that the average number of meals shared by men 35-44 in the 12 months before the interview is equal to the average number of meals shared by dead men aged 35-44 in the 12 months before they died. If the size of people's networks is fixed over time, then Equation I.2 is equivalent to I.1, which we discuss throughout the paper.

We expect that the most common reason for the dynamic decent network condition to fail is that people who are going to die share fewer meals than otherwise similar people who are not about to die (perhaps due to poor health). Ideally, future research would attempt to measure this directly, but even if this measurement does not take place researchers can use the degree ratio parameter in the sensitivity framework ($\delta_{F,\alpha}$) to assess the impact that violating the dynamic decedent network condition would have on death rate estimates (see Section C).

A second possible reason for the dynamic decent network condition to fail is a societal change in the frequency of meal sharing. This issue arises because we learn about meal sharing over two different time periods: for the people who die, we learn about meal sharing in the 12 months before their death and for the respondents, we learn about meal sharing in the 12 months before the interview. For example, suppose an interview was conducted on January 1, 2010 in a country where meal sharing was common in 2009 but there was no meal sharing at all in 2008. We would use the known population method to estimate the respondents' meal sharing during 2009. Now imagine a women who died in the middle of 2009. Half of the year before her death was in the time period where meal sharing never happened. Therefore, the

number of meals she shared in the 12 months before she died (i.e., her degree) will be lower than a women who lived during the entire period. Just as the previous possible concern with the dynamic decedent network assumption, we hope that future work would attempt to measure this possibility directly. But, even if this measurement does not take place, researchers can use the degree ratio parameter in the sensitivity framework ($\delta_{F,\alpha}$) to assess the impact that violating the dynamic decedent network condition would have on death rate estimates (see Section C).

The need to use the dynamic decedent network condition is caused by the tie definition we chose; it is not a property of the network survival estimator generally. If we had used a tie definition that was fixed over time—for example, ties based on a kinship relation (e.g., siblings or cousins) or ties based on mutual attendance at some fixed event—then only the decedent network condition would be needed. Therefore, we consider the trade-off between the decedent network condition and the dynamic decedent network condition to be one of the trade-offs researchers will need to make when considering different tie definitions.

Finally, we note that we designed this specific instrument for our study in Rwanda. Researchers who are interested in applying the network survival method in the future should consider modifying it to account for the context in which they will work. For example, researchers should considering adjusting tie definitions to be more appropriate for their context. Further, if network survival data are collected in a conflict setting, where some respondents may have many connections to people who died, researchers should allow respondents to report more than 12 deaths.

SECTION 2. KNOWN POPULATION

NO.	QUESTIONS AND FILTERS	CODING CATEGORIES	SKIP
200	Now I am going to ask you some questions about people that you know. These questions will help us count the number of people who may be in need of certain health services These people should be:<br>- people you know by sight AND name, and who also know you by sight and name. In other words, you should not consider famous people that you know about, but who do not know about you.<br>- people you have shared a meal or drink with in the past 12 months. These could be family members, friends, co-workers, or neighbors. You should include meals or drinks taken at any location, such as at home, at work, or in a restaurant.<br>- people of all ages who live in Rwanda.		
201	How many men have you shared a meal or drink with whose wife has died and they have not remarried?<br>IF DOES NOT KNOW ANY, RECORD '00'<br>IF KNOWS 95 OR MORE, RECORD '95	NUMBER OF MEN WHOSE WIFE HAS DIED	
202	How many people have you shared a meal or drink with who are currently nurses or doctors?<br>IF DOES NOT KNOW ANY, RECORD '00'<br>IF KNOWS 95 OR MORE, RECORD '95	NUMBER OF NURSES/DOCTORS	
203	How many people have you shared a meal or drink with who are currently male community health workers in 2010?<br>IF DOES NOT KNOW ANY, RECORD '00'<br>IF KNOWS 95 OR MORE, RECORD '95	NUMBER OF MALE COM. HEALTH WORKERS . . .	
204	How many people have you shared a meal or drink with who are currently primary or secondary teachers?<br>IF DOES NOT KNOW ANY, RECORD '00'<br>IF KNOWS 95 OR MORE, RECORD '95	NUMBER OF TEACHERS	
205	How many women have you shared a meal or drink with who currently smoke a pipe or cigarettes?<br>IF DOES NOT KNOW ANY, RECORD '00'<br>IF KNOWS 95 OR MORE, RECORD '95	NUMBER OF WOMEN WHO SMOKE	
206	How many men have you shared a meal or drink with who are currently catholic priests?<br>IF DOES NOT KNOW ANY, RECORD '00'<br>IF KNOWS 95 OR MORE, RECORD '95	NUMBER OF PRIEST	
207	How many people have you shared a meal or drink with who are currently civil servants?<br>IF DOES NOT KNOW ANY, RECORD '00'<br>IF KNOWS 95 OR MORE, RECORD '95	NUMBER OF CIVIL SERVANTS	
208	How many women have you shared a meal or drink with who gave birth in the last 12 months?<br>IF DOES NOT KNOW ANY, RECORD '00'<br>IF KNOWS 95 OR MORE, RECORD '95	NUMBER OF WOMEN WHO GAVE BIRTH	
209	How many people have you shared a meal or drink with who are Muslims?<br>IF DOES NOT KNOW ANY, RECORD '00'<br>IF KNOWS 95 OR MORE, RECORD '95	NUMBER OF MUSLIMS	
210	How many people have you shared a meal or drink with who are currently incarcerated?<br>IF DOES NOT KNOW ANY, RECORD '00'<br>IF KNOWS 95 OR MORE, RECORD '95	NUMBER OF PEOPLE INCARCERATED	
211	How many people have you shared a meal or drink with who were Gacaca judges in 2010?<br>IF DOES NOT KNOW ANY, RECORD '00'<br>IF KNOWS 95 OR MORE, RECORD '95	NUMBER OF GACACA JUDGES	
212	How many men have you shared a meal or drink with who are divorced or separated and not remarried?<br>IF DOES NOT KNOW ANY, RECORD '00'<br>IF KNOWS 95 OR MORE, RECORD '95	NUMBER OF MEN DIVORCED/SEPARATED . . .	

NO.	QUESTIONS AND FILTERS	CODING CATEGORIES	SKIP
213	How many people have you shared a meal or drink with who are being treated for TB?<br>IF DOES NOT KNOW ANY, RECORD '00'<br>IF KNOWS 95 OR MORE, RECORD '95	NUMBER OF PEOPLE<br>TREATED FOR TB	
	Just as a reminder I am only interested in<br>- people you shared a meal or drink with in the past 12 months<br>- People of all ages who live in Rwanda.		
214	How many people have you shared a meal or drink with are named NSENGIMANA?<br>IF DOES NOT KNOW ANY, RECORD '00'<br>IF KNOWS 95 OR MORE, RECORD '95	NUMBER OF<br>NSENGIMANA	
215	How many people have you shared a meal or drink with are named MUREKATETE?<br>IF DOES NOT KNOW ANY, RECORD '00'<br>IF KNOWS 95 OR MORE, RECORD '95	NUMBER OF<br>MUREKATETE	
216	How many people have you shared a meal or drink with are named TWAHIRWA?<br>IF DOES NOT KNOW ANY, RECORD '00'<br>IF KNOWS 95 OR MORE, RECORD '95	NUMBER OF<br>TWAHIRWA	
217	How many people have you shared a meal or drink with are named MUKANDEKEZI?<br>IF DOES NOT KNOW ANY, RECORD '00'<br>IF KNOWS 95 OR MORE, RECORD '95	NUMBER OF<br>MUKANDEKEZI	
218	How many people have you shared a meal or drink with are named NSABIMANA?<br>IF DOES NOT KNOW ANY, RECORD '00'<br>IF KNOWS 95 OR MORE, RECORD '95	NUMBER OF<br>NSABIMANA	
219	How many people have you shared a meal or drink with are named MUKAMANA?<br>IF DOES NOT KNOW ANY, RECORD '00'<br>IF KNOWS 95 OR MORE, RECORD '95	NUMBER OF<br>MUKAMANA	
220	How many people have you shared a meal or drink with are named NDAYAMBAJE?<br>IF DOES NOT KNOW ANY, RECORD '00'<br>IF KNOWS 95 OR MORE, RECORD '95	NUMBER OF<br>NDAYAMBAJE	
221	How many people have you shared a meal or drink with are named NYIRANEZA?<br>IF DOES NOT KNOW ANY, RECORD '00'<br>IF KNOWS 95 OR MORE, RECORD '95	NUMBER OF<br>NYIRANEZA	
222	How many people have you shared a meal or drink with are named BIZIMANA?<br>IF DOES NOT KNOW ANY, RECORD '00'<br>IF KNOWS 95 OR MORE, RECORD '95	NUMBER OF<br>BIZIMANA	
223	How many people have you shared a meal or drink with are named NYIRAHABIMANA?<br>IF DOES NOT KNOW ANY, RECORD '00'<br>IF KNOWS 95 OR MORE, RECORD '95	NUMBER OF<br>NYIRAHABIMANA	
224	How many people have you shared a meal or drink with are named NDAGIJIMANA?<br>IF DOES NOT KNOW ANY, RECORD '00'<br>IF KNOWS 95 OR MORE, RECORD '95	NUMBER OF<br>NDAGIJIMANA	
225	How many people have you shared a meal or drink with are named MUKANDAYISENGA?<br>IF DOES NOT KNOW ANY, RECORD '00'<br>IF KNOWS 95 OR MORE, RECORD '95	NUMBER OF<br>MUKANDAYISENGA	

NO.	QUESTIONS AND FILTERS	CODING CATEGORIES		SKIP
226	Now I would like to ask you a few questions about people who have died.<br>Similar to the previous questions only tell me about<br>- people you shared a meal or drink with in the past 12 months before they died.<br>- These should be people of all ages living in Rwanda.<br><br>How many people have you shared a meal or drink with who have died in the past 12 months?	NUMBER OF DEATHS . . . . [ ][ ]<br><br>NONE 00		→ 301
227	I would like to ask a couple of questions about each of these people who died. To keep track of the different people we are discussing, could you tell me the first name of each person you know who died in the past 12 months?<br>RECORD THE FIRST NAME OF EACH PERSON WHO HAS DIED AND ASK Q.228 AND 229<br><br>IF AGE IS NOT KNOWN, GET THE BEST POSSIBLE ESTIMATE<br>IF AGE 95 OR MORE, RECORD '95'	228<br>Was (NAME) male or female?	229<br>How old was (NAME)?	
	NAME 1 ________________	MALE . . . . 1<br>FEMALE 2	[ ][ ]	
	NAME 2 ________________	MALE . . . . 1<br>FEMALE 2	[ ][ ]	
	NAME 3 ________________	MALE . . . . 1<br>FEMALE 2	[ ][ ]	
	NAME 4 ________________	MALE . . . . 1<br>FEMALE 2	[ ][ ]	
	NAME 5 ________________	MALE . . . . 1<br>FEMALE 2	[ ][ ]	
	NAME 6 ________________	MALE . . . . 1<br>FEMALE 2	[ ][ ]	
	NAME 7 ________________	MALE . . . . 1<br>FEMALE 2	[ ][ ]	
	NAME 8 ________________	MALE . . . . 1<br>FEMALE 2	[ ][ ]	
	NAME 9 ________________	MALE . . . . 1<br>FEMALE 2	[ ][ ]	
	NAME 10 ________________	MALE . . . . 1<br>FEMALE 2	[ ][ ]	
	NAME 11 ________________	MALE . . . . 1<br>FEMALE 2	[ ][ ]	
	NAME 12 ________________	MALE . . . . 1<br>FEMALE 2	[ ][ ]	
